# Bacteria-Infected Artificial Urine Characterization Based on a Combined Approach Using an Electronic Tongue Complemented with ^1^H-NMR and Flow Cytometry

**DOI:** 10.3390/bios13100916

**Published:** 2023-10-05

**Authors:** Carolin Psotta, Emelie J. Nilsson, Thomas Sjöberg, Magnus Falk

**Affiliations:** Biomedical Science, Faculty of Health and Society, and Biofilms Research Center, Malmö University, 205 06 Malmö, Sweden; carolin.psotta@mau.se (C.P.); emelie.nilsson@mau.se (E.J.N.); thomas.sjoberg@mau.se (T.S.)

**Keywords:** electronic tongue, bacterial detection, artificial urine, urinary tract infection, ^1^H-NMR, flow cytometry

## Abstract

The prevailing form of bacterial infection is within the urinary tract, encompassing a wide array of bacteria that harness the urinary metabolome for their growth. Through their metabolic actions, the chemical composition of the growth medium undergoes modifications as the bacteria metabolize urine compounds, leading to the subsequent release of metabolites. These changes can indirectly indicate the existence and proliferation of bacterial organisms. Here, we investigate the use of an electronic tongue, a powerful analytical instrument based on a combination of non-selective chemical sensors with a partial specificity for data gathering combined with principal component analysis, to distinguish between infected and non-infected artificial urine samples. Three prevalent bacteria found in urinary tract infections were investigated, *Escherichia coli*, *Klebsiella pneumoniae*, and *Enterococcus faecalis*. Furthermore, the electronic tongue analysis was supplemented with ^1^H NMR spectroscopy and flow cytometry. Bacteria-specific changes in compound consumption allowed for a qualitative differentiation between artificial urine medium and bacterial growth.

## 1. Introduction

Urinary tract infection (UTI) is the most frequently occurring bacterial infection and is caused by microorganisms which access the urinary tract [1,2]. While *Escherichia coli* causes the majority of bacterial infections, a large variety of pathogenic bacteria exists [1,2,3,4]. This includes both Gram-negative species such as *Proteus mirabilis*, *Proteus vulgaris*, *Klebsiella pneumoniae*, and *Pseudomonas aeruginosa*, and Gram-positive species such as *Enterococcus faecalis*, *Streptococcus agalactiae*, *saprophyticus* and *aureus* [2,3,5,6,7]. A majority of infections resolve without complications; however, serious progression can lead to bacteremia, sepsis or even death, making fast identification of infection important [1,6]. Typically, diagnosis of UTI involves assessing clinical symptoms and confirming results through laboratory and microbiological tests (such as colony counting and PCR or ELISA detection), a time- and cost-consuming process that can take several days to confirm the presence of infection [1,3,7,8,9]. As an alternative to such laboratory-based methods, point-of-care (POC) testing offers significant benefits for both patient care and diagnosis, with the possibility of rapid, cost-effective, and patient-near detection of different diseases or infections [10,11,12,13,14,15]. In this regard, the development of label-free bacterial sensors with rapid response times, capable of both qualitative and quantitative detection of bacteria within the clinical standard range (10^5^ CFU mL^−1^ for UTI), holds paramount significance [1,3,16,17].

Instead of relying on the immobilization of biorecognition elements specific for one type of bacteria for bacterial detection, an alternative approach is to utilize metabolic (bio)markers related to the growth of bacteria. Upon accessing the urinary tract, bacteria exploit the human urine metabolome for their proliferation. Urine constitutes a dilute mixture with varying concentrations of nutrients for bacteria, including urea, creatinine, amino acids, acids (such as lactic acid and citric acid) serving as carbon, nitrogen and hydrogen sources, alongside different ions like sodium or potassium [4,18,19,20]. In comparison to typical growth conditions, the primary sources for bacterial growth in urine differ, as normal urine contains only small amounts of glucose [21]. As bacteria grow, they both consume and release a variety of small molecules, leading to a change in the chemical composition of their surroundings [20,22,23]. This change can be harnessed for bacterial detection, as certain metabolites, such as acetate, lactate, succinate, and formate, are exclusive to infected urine [23,24]. Monitoring this change can facilitate the early-stage diagnosis of UTIs [16,25].

Detecting variations in bacterial metabolites within multi-component solutions, such as urine, poses significant challenges for sensor development. However, one promising approach to overcome these challenges is by utilizing electrochemical sensing with a combination of different low-selective electrodes combined with signal processing based on Pattern Recognition and/or Multivariate data analysis, inspired by the human sense of taste and commonly referred to as electronic tongues (e-tongues) [26,27]. Employing an e-tongue concept allows for recording of partially overlapping but still specific selectivity patterns for different electrode materials upon changing analyte compositions which can be separated upon further processing, providing a unique ability to deal with a complex and changing background and diminish the impact of interferents [28,29,30]. Collecting analyte-specific responses with, for example, voltammetric techniques (e.g., differential pulse voltammetry), the sensor response for each sample can be arranged in one data set where the individual electrode response for each electrode in the sensor array, e.g., recorded current, makes up the combined response for each individual sample. The data set can then be further analyzed with data-processing algorithms and pattern recognition methods [26,28,29,30,31]. Principal component analysis (PCA) is commonly used to analyze the response of an e-tongue, which is a method to reduce (big) data sets without losing essential information [28,29,30,32,33]. This approach facilitates the analysis of complex multi-component fluids like juice, milk, drinks, food and flavors [28,34], and has also recently been used to analyze physiological fluids like virus detection in saliva [35], oral cavity cancer diagnostic in saliva [36], compositional change in sweat [37], urea, creatinine, and ion detection in dialysate fluid [38]. While some efforts have been made to deploy e-tongues for urine analysis, such as different potentiometric electronic tongues for ion analysis as well as the possible use in cancer diagnosis, as far as we know, no studies have been made using electronic tongues for UTI classification [39,40,41,42].

Herein, we present an electrochemical, label-free detection method for bacteria based on the different electrochemical responses caused by chemical changes induced by bacterial growth in artificial urine medium (AUM). AUM was used to mimic real urine, enabling a more realistic and detailed examination of bacterial growth compared to common growth media [5]. The detection method was established by assembling an e-tongue composed of three different electrode materials, viz., platinum, palladium and gold. The e-tongue was operated in AUM and used to monitor the growth of three different bacterium types all associated with UTI, two Gram-negative and one Gram-positive bacterium, *Escherichia coli* (*E. coli*), *Klebsiella pneumoniae* (*K. pneumoniae*), and *Enterococcus faecalis* (*E. faecalis*), respectively. Combining the e-tongue with ^1^H-NMR provided a qualitative analysis of the bacterial metabolic activity.

## 2. Materials and Methods

### 2.1. Chemicals and Materials

Artificial urine medium (AUM) was prepared according to Table 1 and adjusted to a pH of 6.4 with NaOH or HCl, according to a previously established protocol [43]. Magnesium sulfate was purchased from Kebo Lab AB (Stockholm, Sweden). Peptone from meat (bacteriological), lactic acid, citric acid, uric acid, urea, creatinine, calcium chloride, iron chloride and sodium chloride were all purchased from Sigma Aldrich (Merck KGaA, St. Louis, MI, USA). Yeast extract, sodium bicarbonate, ammonium chloride, di-potassium hydrogen phosphate and sodium sulfate were all purchased from Merck (St. Louis, MI, USA). Potassium dihydrogen phosphate was purchased from Duchefa Biochemie (Haarlem, The Netherlands). All solutions were prepared with the MilliQ-water with 18.2 MΩ cm^−1^ resistivity.

### 2.2. Bacterial Culture

To inoculate single bacterial colonies, *E. coli* (ATCC25922), *K. pneumoniae* (ATCC) and *E. faecalis* (ATC29212) were grown on sterile lysogeny broth (LB) agar plates. For each experiment, one colony of the bacterial species of interest was taken and grown in 40 mL AUM overnight in the incubator at 37 °C. Before each measurement, quantitative bacterial analysis was performed with a spectrophotometer (Pharmaspec UV-1700 spectrophotometer, Shimadzu, Kyoto, Japan) by measuring the transmission at 600 nm. The optical density measurements were used to estimate the number of cells per ml (OD_600_ = 1.0 = 8 × 10^8^ cells mL^−1^) [44].

### 2.3. Sensor Preparation

Gold (d = 2 mm), palladium (d = 3 mm), and platinum (d = 1.6 mm) electrodes were mechanically cleaned with Alumina polishing paste on a micro cloth (Buehler, IL, USA) for 3 min on a cloth pellet and rinsed with MilliQ water. Afterwards, the gold and palladium electrodes were electrochemically cleaned by cycling in 0.5 M H_2_SO_4_ from −0.2–1.7 V with a scan rate of 100 mV/s for 20 cycles. The platinum electrode was electrochemically cleaned by cycling in 0.5 M H_2_SO_4_ from −0.5–1.2 V with a scan rate of 100 mV s^−1^ for 10 cycles.

### 2.4. Electrochemical Measurements

All measurements were performed with a μAutolab Type III/FRA2 potentiostat/galvanostat from Metrohm Autolab B.V. (Utrecht, The Netherlands) equipped with a GPES software version 4.9 using differential pulse voltammetry (DPV) with the following parameters: a pulse amplitude of 0.02505 V, a step potential of 0.00195 V, and a potential range of −0.5–+1.4 V, recording 958 individual data points for each DPV measurement. All measurements were performed in a three-electrode set up with an Ag|AgCl|3 M KCl reference electrode, a platinum wire counter electrode, using one of the working electrodes (gold, palladium, or platinum) at a time. For each measurement, a beaker containing 55 mL AUM was connected to a water bath to maintain a constant temperature of 37 °C while continuously stirring. After reaching 37 °C, the bacterial aliquots were introduced. The added aliquot volumes were based on the measured transmission of the overnight culture. Herein, the aimed starting concentration for each species was 10^5^ CFU mL^−1^ since this is the clinically relevant cut-off value for UTI [1,3]. For *K. pneumoniae*, aliquots between 300–400 µL were taken (T % = 68.6 ± 3.2%; *n* = 4), for *E. faecalis* 400 µL (T % = 74.7 ± 4.5%: *n* = 4) and for *E. coli* 400 µL (T % = 72.3 ± 1.8%; *n* = 4). The beaker was kept closed until measurements were performed when it was opened to allow room for the electrodes. For the initial characterization, measurements were performed in only AUM and after 5 h with bacteria (without interruption of the growing process). When combined with flow cytometry, the bacterial aliquot was added and DPVs were then recorded at 0, 1, 2, 3, 4, and 5 h of incubation time, where the beaker was closed after each measurement. After taking the last aliquot at 5 h incubation time for the flow cytometry measurements, the analysis of the supernatant followed. For this purpose, the bacterial solution after a 5 h incubation time was transferred into tubes and spun down with a centrifuge at 5000 rpm (2000× *g*) for 5 min. Afterwards, the supernatant was taken without the bacterial pellet and investigated using DPV.

### 2.5. ^1^H NMR Spectroscopy

Artificial urine samples were collected from the overnight culture from the three investigated bacteria species. To avoid bacterial contamination, a 500 μL aliquot was spun down with a centrifuge at 5000 rpm (2000× *g*) for 5 min and the supernatant was taken without the bacterial pellet. The supernatant of each sample was transferred in 1 mL plastic Eppendorf tubes and left for evaporation until dry under vacuum (GeneVac centrifugal evaporator EZ-2, SP Scientific). The dried samples were then redissolved in D_2_O prior to the measurements to allow the spectrometer to find a lock signal by adding 550 µL of D_2_O to the Eppendorf, vortexing it, leaving in an ultrasonic bath for 60 min and vortexing again before transferring 500 µL to a 5 mm onetime-use NMR tube. The NMR spectra were collected on a Varian Mercury 400 MHz spectrometer at a resonance frequency of 400.41 MHz using a 5 mm Varian 400 ASW 1H/13C/31P/15N/4NUC PFG 40–162 MHz (SN40P5A910) probe at 25 °C. The spectra were acquired with the following parameters: a 90 °C pulse (11.5 μs pulse width), relaxation delay of 5 s, acquisition time of 2.6 s, a spectral width of 6406.1 Hz (−3.3 to 12.7 ppm), with 16,384 complex data points, and a 20 Hz spin. For each spectrum, 256 scans were collected. All spectra were Fourier-transformed using MestReNova (version 14.1.2, Mestrelab Research, Escondido, CA, USA), including phasing and baseline correction. The area of the peaks was calculated by fitting Lorentzian–Gaussian peaks to the regions of interest. The chemical shift of creatinine at 4.05 ppm was used as the reference peak.

### 2.6. Flow Cytometry

To monitor the growth of *E. coli*, *K. pneumoniae* and *E. faecalis* in AUM, flow cytometry was performed using a dsDNA quantitation kit (Pico488 from Lumiprobe GmbH, Hannover, Germany). Briefly, 1 mL of undiluted AUM was collected at different time intervals from the electrochemical cell and centrifuged at 5000 rpm (2000× *g*) for 5 min. An amount of 950 µL of the supernatant was removed and the remaining solution was resuspended in 150 µL 1:200 SYBR Green (Pico488 from Lumiprobe GmbH, Germany) and 150 µL staining buffer (phosphate-buffered saline containing 0.01% Tween-20 and 1 mM ethylenediaminetetraacetic acid (EDTA)), incubated for 10 min at 37 °C. Next, a fixed volume (25 µL) of stained bacteria was analyzed with the flow cytometer (BD Accuri™ CG plus from Becton Dickinson, East Rutherford, NJ, USA) using a low sample rate and a selected threshold setting on FSC (Forward-scatter) and SSC (Side-scatter). An unstained sample was used to gate the bacteria since SYBR Green only binds to DNA and no other compounds in the AUM. The amount of SYBR Green-stained bacteria/cells per ml could be calculated by multiplying the number of stained bacteria detected in the dot plot by the conversion factor of 14. Aliquots for the analysis were taken at the specific incubation times of 0, 1, 2, 3, 4, and 5 h (Figures S1–S3, Supplementary Materials). The protocol for counting bacteria in AUM was based on a previously described method by Moshaver et al. [45].

### 2.7. Data Treatment with Principal Component Analysis in SPSS

PCA was performed using SPSS Statistics Ver. 28.0.1.1 from IBM Corp. (Armonk, NY, USA) as an unsupervised dimension reduction tool to analyze the voltammetric responses qualitatively. Prior to analysis, the data sets for responses from the three working electrodes were merged into one data set and analyzed together with each repeated measurement, with the responses standardized to remove the effects of different electrode sizes by having each variable (response from each electrode) scaled to unit variance and mean-centered. After pre-processing, PCA of samples was performed, extracting all factors with an eigenvalue greater than 1 without any additional factor rotation.

## 3. Results and Discussion

### 3.1. Charachterization of Bacterial Growth in AUM

To investigate the different bacteria, specifically *K. pneumoniae*, *E. faecalis*, and *E. coli* and their growth behavior in AUM, three electrode materials were employed, viz. Pt, Pd, and Au. These materials were chosen as the noble metals provide a simple and robust sensing platform while having different catalytic properties, leading to different response profiles [26]. While the use of traditional noble metal electrodes would be expensive in a practical application, screen-printing processes performed on polymeric substrates allow for fast and cost-efficient mass production of miniaturized, single-use electrochemical sensors [46].

**Figure 1:**
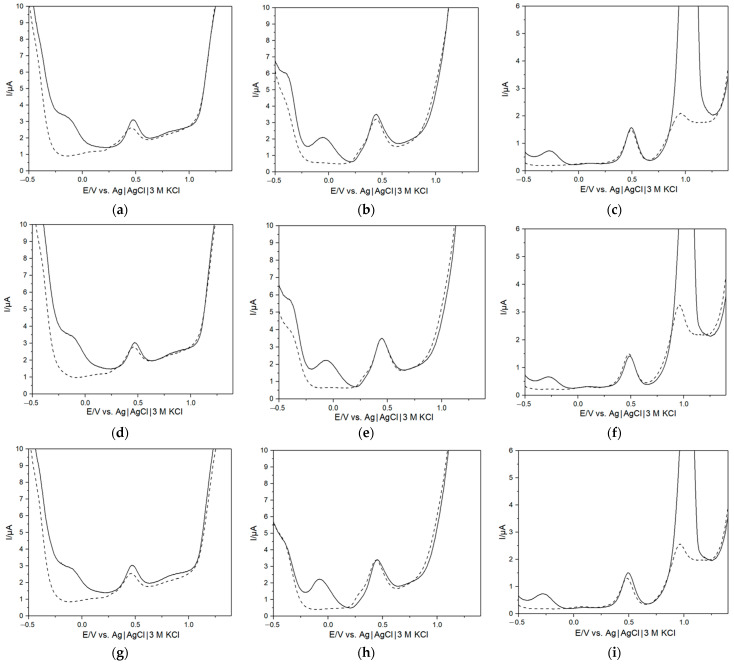
Differential pulse voltammograms (solid = only AUM; dashed = after 5 h incubation) of *K. pneumoniae* (**a**–**c**)*, E. faecalis* (**d**–**f**) and *E. coli* (bottom row) using a Pt electrode (left column), Pd electrode (middle column) and a Au electrode (**g**–**i**).

For the initial characterization of the e-tongue, bacteria were added to AUM and the response was measured after 5 h. The added aliquot volumes targeted a starting concentration of roughly 10^5^ CFU mL^−1^ in order to be close to the clinically relevant cut-off value for UTI as well as to balance providing an adequate number of viable, active cells to track metabolic changes while mitigating the risk of transferring excessive toxins and providing insufficient substrate. Typical DPVs for the three electrode materials and the three bacteria species are presented in Figure 1a–i (solid = only AUM; dashed = after 5 h incubation). The general electrochemical behavior of noble metal electrodes at neutral pH is well studied and understood, where large currents at minimal and maximal redox potentials could be attributed to H_2_ adsorption/evolution as well as O_2_ reduction reactions and H_2_O electro-oxidation, respectively [47,48,49,50,51,52].

While general shifts in peaks can be attributed to pH changes during bacterial growth, resulting from the utilization of compounds in the AUM or the release of metabolites, consequently altering the chemical composition of the medium [20,22,23,53], the investigation of all bacterial species revealed significant changes in two potential ranges: −0.35 to 0.2 V and 0.8 to 1.0 V. The main compounds used for bacterial growth in AUM (Table 1) are urea, ammonia, and peptone (necessary gluconeogenic amino acids) which also serve as sources of nitrogen; additionally, carbon is primarily derived from peptone, lactic acid and citric acid [7,9,33]. In order to try to understand the changes in peak characteristics resulting from bacterial metabolism, it was necessary to relate the growth-relevant AUM compounds to the recorded peaks in the voltammograms. The AUM composition is complex and has several electrochemically active compounds in the investigated potential range; however, certain regions could be identified and related to specific growth compounds (Figure S4, Supplementary Materials). Specifically, uric acid oxidation occurred at potentials around +0.4 V for the Pt (Figure 1a,d,g) [54] and Pd (Figure 1b,e,h) electrodes [55] and around +0.45 V for the Au (Figure 1c,f,i) electrode [56]. However, while some changes were observed in the peak in repeat measurements, it was inconsistent and could not be directly attributable to bacterial growth. In addition, compared to the Pt and Pd electrodes, for the Au electrode, a clear peak at +1.0 V was observed related to electrooxidation of the growth compounds at potentials ≥+1.0 V on gold surfaces [57,58]. After 5 h of bacterial growth, a significant drop was observed, attributable to consumption of growth compounds during the bacterial metabolism. Furthermore, a noteworthy difference was also observed when comparing measurements in AUM to bacterial growth in the low potential region, attributable to H_2_ evolution and O_2_ reduction, which was significantly diminished. A possible explanation could be that compounds produced due to the bacterial metabolism may adsorb on the surface and change the electrocatalytic properties of these materials. Similar behavior was recently observed when saliva samples were investigated [35]. To confirm that the differences in the voltammograms were based on altered bacterial metabolism and its redox-active behavior rather than from cell adsorption, the supernatant from all three bacterium types was also electrochemically investigated (Figure S5, Supplementary Materials), where a similar behavior was observed as with bacterial cells in solution [59].

**Figure 2:**
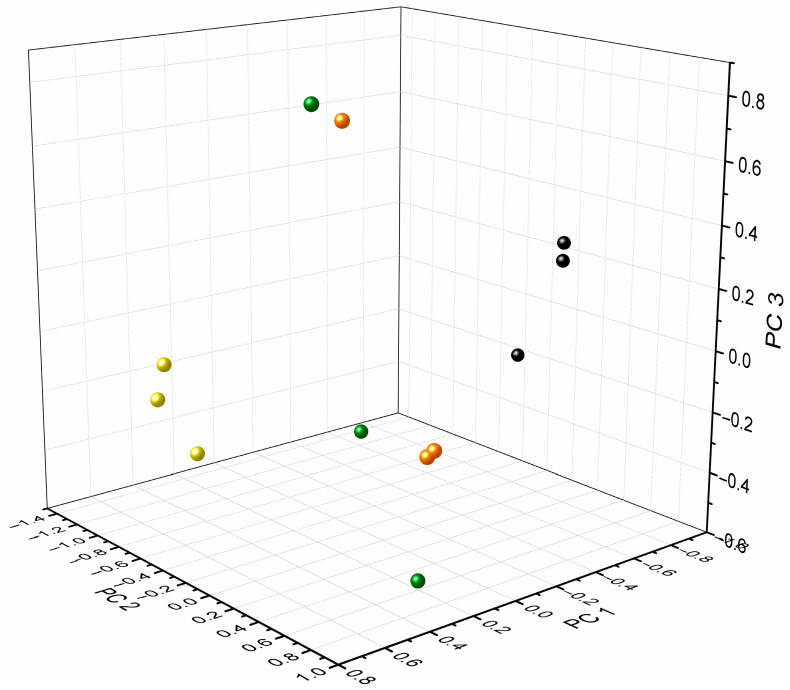
PCA score plot comparing AUM (black) and bacteria-infected AUM (*K. pneumoniae* (yellow), *E. faecalis* (green), *E. coli* (orange)); explained variance of PC 1 (36.7%), PC 2 (21.5%), and PC 3 (16.3%).

While significant disparities were evident in the voltammograms of infected and non-infected solutions, variations between distinct species were very subtle. To investigate the possibility to differentiate different types of bacteria, a large data set of 12 × 2874 (34,488 data points) was combined, including measurements with all three electrode materials in AUM (*n* = 3), *K. pneumoniae* (*n* = 3), *E. faecalis* (*n* = 3), and *E. coli* (*n* = 3). In Figure 2, the related PCA score plot is presented and compares AUM (black) with *K. pneumoniae* (yellow), *E. faecalis* (green), and *E. coli* (orange) (score values, Table S1, Supplementary Materials). Overall, four PCs were extracted which explained a total variance of 78.7%, specifically PC 1 = 36.7%, PC 2 = 21.5%, PC 3 = 16.3% and PC 4 = 14.3%. All bacteria were well distinguishable from AUM, mainly separated on PC 1. Interestingly, for the analysis of *K. pneumoniae* a clear data clustering was observed compared to the other two bacteria species. This could possibly be related to bacteria-specific differences in the metabolism, e.g., usage and utilization of different artificial urine compounds and morphological characteristics (rod vs. coccus-shaped) which could lead to these growth behavior variations [5,24,60,61]. It is also known that adaptations in essential metabolic pathways for bacteria occur to enable growth in urine (e.g., amino acid catabolism, gluconeogenesis, and TCA cycle) [18].

### 3.2. Quantitative Analysis of Bacterial Growth

**Figure 3:**
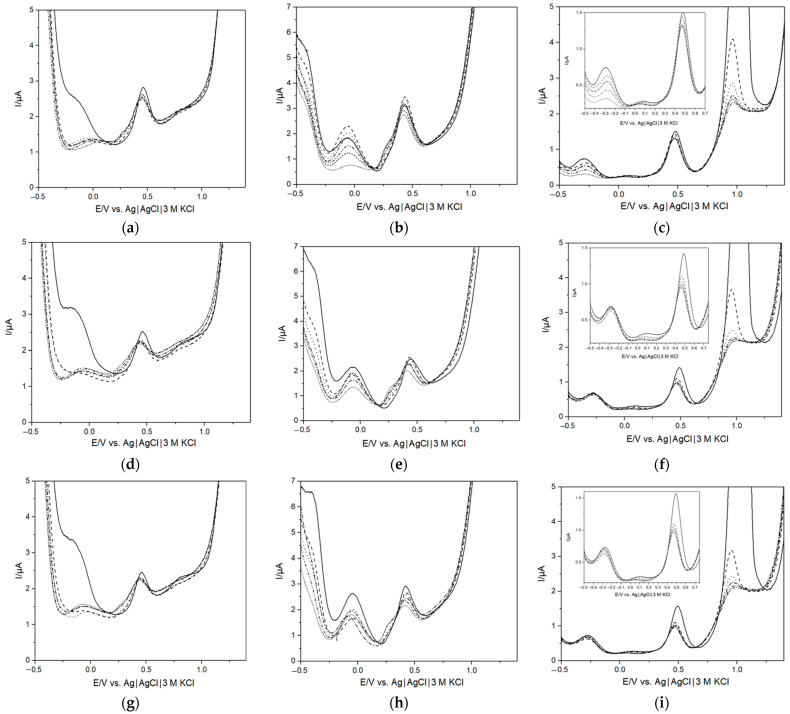
Differential pulse voltammograms taken hourly (solid = AUM; 1 h = dashed; 2 h = dotted; 3 h = dash dot; 4 = dash dot dot; 5 h = short dotted) of *K. pneumoniae* (**a**–**c**), *E. faecalis* (**d**–**f**) and *E. coli* (**g**–**i**) using a Pt electrode (left column), Pd electrode (middle column) and a Au electrode (right column).

In order to determine how the growth of bacteria could be related to the response of the e-tongue, measurements were combined with flow cytometry to see if a relationship between the number of bacteria and the response of the e-tongue could be established. Hourly measurements were performed to enable precise tracking of the compositional changes in the AUM caused by bacterial proliferation. The results are shown in Figure 3, and the corresponding bacterial concentrations, as measured by flow cytometry, are presented in Table 2. The initial measurement at 0 h, immediately following the addition of the bacterial aliquot, was excluded due to the absence of discernible discrepancies in voltammograms when compared to AUM. A gradual decline in measured currents was observed across both the low-potential and high-potential regions as the duration of bacterial presence in the solution increased. As discussed above (Section 3.1), the changes in the response of the e-tongue can be related to the consumption of AUM compounds and the release of metabolites. Importantly, significant changes occurred within the first hour associated with bacterial growth, within the clinically relevant range of 10^5^ CFU mL^−1^ [1,3,16,17] for each investigated bacteria species.

Based on the results from the flow cytometry, clear differences were observed in the growth of the different species (Table 2). All aliquots were taken out of the stationary phase of the overnight culture. While *K. pneumoniae* entered into a logarithmic growth phase after a few hours, this was not observed for *E. faecalis* and *E. coli,* which displayed minor proliferation where *E. coli* then sustained the cell number the following hours and *E. faecalis* rather displayed minor cell death/decline. The different growth behavior can be explained by individual and metabolic growth adaptations in AUM where bacteria adjust to the new surroundings before transitioning from the lag-phase to the log-phase [62]. The observed differences in bacterial growth between the species could possibly be based on the ability of *K. pneumoniae* to utilize urea (production of urease). According to the microbiological urease test, *E. faecalis* and *E. coli* are generally considered “urease-negative” and thus unable to break down urea into ammonia and CO_2_ [63,64]. The produced ammonia could be utilized as a nitrogen source for growth [19,65]. Additionally, the distinct morphologies of coccus-shaped and rod-shaped bacteria contribute to their diverse growth behaviors and characteristics [66]. The difference in growth behavior could explain the observed separation of *K. pneumoniae.* from the other bacteria as seen in Figure 3a–c. It should be noted that the behavior of *K. pneumoniae.* and *E. faecalis* was similar as to what was observed when growth curves of each of the species were recorded (Figure S6, Supplementary Materials); however, a clear difference in behavior was observed for *E. coli* where a typical log-phase behavior was observed. The different behavior could be explained by the higher starting volumes of the overnight cultures used, where a thousand-fold difference in starting concentration was used for the flow cytometry in Table 2 compared to Supplementary Materials Figure S6. It is well known that bacterial proliferation is highly dependent on the starting conditions, including cell density.

When comparing Figure 1 to Figure 3, a distinct difference could be observed between the recorded voltammograms for *E. coli* (g)–(i) and *E. faecalis* (d)–(f). The results from measurements after 5 h (Figure 1) showed a clear decrease in the low-potential region, which was not observed when measurements were taken hourly. In contrast, the response demonstrated by *K. pneumoniae* (a)–(c) remained notably consistent. One difference between the species was that while *K. pneumoniae* were growing very well in AUM, the growth of the other species was somewhat retarded. Plausible reasoning for this divergence could potentially be attributed to subtle experimental variations. Notably, the periodic opening of the container for e-tongue measurements and aliquot extraction for flow cytometry, undertaken hourly, could have perturbed the growth behavior. This is in contrast to the uninterrupted growth experienced over the initial 5 h period.

**Figure 4:**
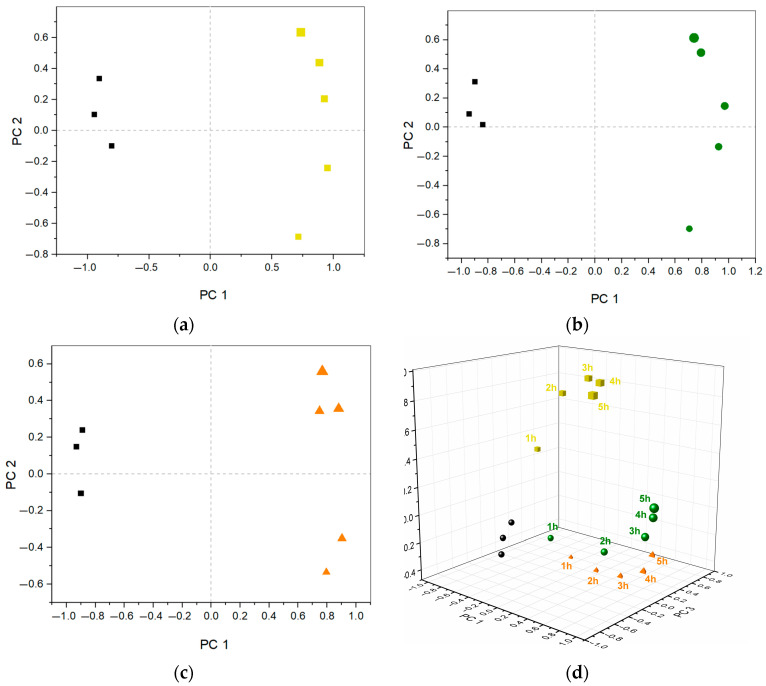
(**a**–**c**) PCA score plots for AUM (black) and at different bacterial incubation times (1, 2, 3, 4, 5 h—increasing symbol size corresponding to increasing time) of *K. pneumoniae* ((**a**), yellow square), *E. faecalis* ((**b**), green circle) and *E. coli* ((**c**), orange triangle). (**d**) Combined 3D-score plot comparing AUM and all bacteria-infected samples at different incubation times, explained variance of PC 1 (37.4%), PC 2 (23.2%), and PC 3 (20.8%).

To elucidate the distinctive response recorded by the e-tongue across various growth stages, PCA was employed. To carry this out, the data from each of the three electrodes recorded on one experimental day from a single species at 1, 2, 3, 4, and 5 h incubation time with three repeat measurements of AUM was combined into three separate data sets of 8 × 2874 data points. The results are shown in Figure 4a–c, where two different principal components (PCs) were extracted for each of the species, all with absolute Eigenvalues greater than one, explaining a total variance of roughly 90% in all three cases, with PC 1 explaining around 74% and PC2 around 16% (score values, Tables S2–S4, Supplementary Materials). A similar trend was observed for all three bacterial species, with a gradual increase in the loading of PC 2 the longer the incubation time in AUM. While in the case of *K. pneumoniae* this change could be related to increasing bacterial concentration, this was not the case for the other species. Instead, the observed trend was primarily linked to growth duration, implying the consumption of growth medium and subsequent release of metabolites, rather than fluctuations in bacterial concentration.

In addition, the separate bacterial species were analyzed together using a combined data set of 18 × 2874 data points. In total, four different principal components (PCs) were extracted, all with absolute Eigenvalues greater than one, explaining a total variance of 90.4% in the samples, where 37.4%, 23.2%, 20.8% and 9.0% were explained by PC 1–4, respectively (score values, Table S5, Supplementary Materials). The resulting score plot is shown in Figure 4d, where AUM is shown in black spheres with the response from the bacterial growth over the incubation period of 1, 2, 3, 4, and 5 h (increasing symbol size) for *K. pneumoniae* ((**a**), yellow cube), *E. faecalis* ((**b**), green sphere), and *E. coli* ((**c**), orange tetrahedron). Similarly, as observed in Figure 2, AUM samples exhibited distinct segregation from samples containing bacterial growth. Furthermore, *K. pneumoniae* was clearly separated from *E. faecalis* and *E. coli*. In order to further investigate growth trends, a normalized current response based on the whole data set is shown in Supplementary Materials Figure S7, highlighting the differences between 1 h and 5 h incubation time for the different species. Certain regions in the voltammograms can be observed to contribute to sample variations which facilitate differentiation, e.g., a clear change was observed between 1 h and 5 h for *K. Pneumoniae* particularly in the low-potential region of the Au and Pd electrodes. By expanding the analysis to include additional species and leveraging a larger dataset, this could be used to define appropriate measurement parameters by selecting regions for the individual electrodes to improve species classification. Furthermore, increasing the incubation time and/or changing the growth conditions could improve differentiation, particularly as *E. coli* and *E. faecalis* displayed poor proliferation.

### 3.3. Investigation of Metabolic Changes in AUM Due to Bacterial Growth

**Figure 5:**
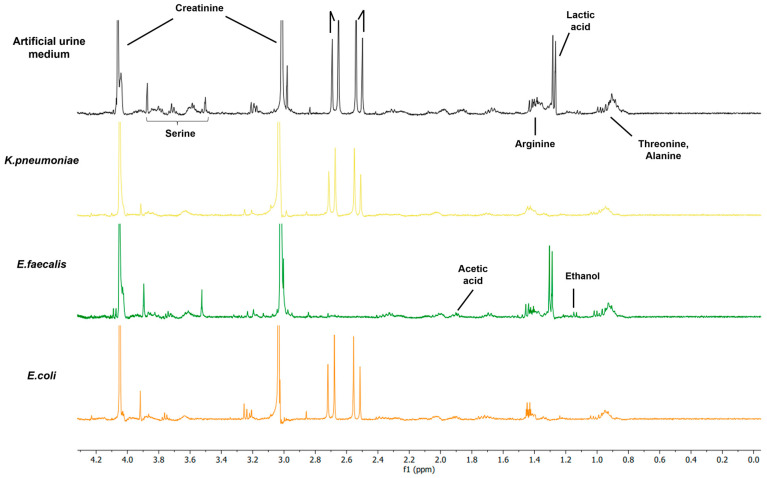
^1^H NMR spectra of AUM (black) and after 5 h of growth of *K. pneumoniae* (yellow), *E. faecalis* (green) and *E. coli* (orange).

In order to allow for the identification of specific components, the electrochemical measurements were supplemented with ^1^H NMR. This complementary approach allowed for a qualitative assessment of the metabolic alterations occurring during bacterial growth within the AUM. The corresponding spectra of AUM (black), *K. pneumoniae* (yellow), *E. faecalis* (green) and *E. coli* (orange) are shown in Figure 5 (Figures S8–S12, Supplementary Materials), and the associated chemical shifts are detailed in Table 3. For the analysis of AUM, characteristic peaks of the main compounds could be identified: amino acids in peptone (e.g., glucogenic amino acids, like threonine and alanine, m, 1.20–1.36 ppm; arginine, d, 1.57, 1.59; serine, m, 3.42–3.84 ppm (Figures S11 and S12, Supplementary Materials); lactic acid (d, 1.31, 1.32 ppm [19,43]), citric acid (d, 2.50, 2.54, 2.65, 2.69 ppm [32,43]), and creatinine (s, 3.03 and 4.05 ppm [32,43,44]). As previously shown in the literature, uric acid is not visible at physiological pH [43]. In the case of creatinine, D_2_O causes a hydrogen-deuterium exchange of the −CH_2_, corresponding to the peak at 4.05 ppm, whereas the −CH_3_ should remain unaffected by the D_2_O (peak at 3.03 ppm) [67]. The aforementioned exchange can easily lead to inaccurate quantification of the creatinine peak. However, the observed decrease in the peak intensities at 3.03 ppm in the bacterial samples compared to the clean AUM control sample indicates a decrease in creatinine levels attributable to bacterial metabolism. In general, reported significant metabolic changes in the chemical composition of the urine due to bacterial growth can be identified as the production of acetic acid (1.93 ppm) [19,32,43,44,45], ethanol (1.17 ppm) [19,45], succinate [45], but also formate (8.3 ppm) [19].

In comparison to the AUM control sample, the growth of *K. pneumoniae* within the AUM medium yielded a notable absence of the lactic acid peak, effectively eliminating the discernible signal at 1.3 ppm. Additionally, the citric acid content witnessed a significant reduction, diminishing by approximately half. Moreover, the chemical shift pattern related to peptone in AUM changed, confirming bacterial growth. Notably, reductions in peak intensity were evident within the chemical shift regions corresponding to threonine, alanine, and serine. In contrast to *K. pneumoniae*, for *E. faecalis* a different behavior was observed; the lactic acid peak remained slightly elevated in comparison to AUM (indicative of lactic acid production). Most importantly, *E. faecalis* entirely depleted the citric acid for metabolic processes. Furthermore, two bacterial metabolites could be identified, viz. acetic acid [15,39] and ethanol [39], prominently observed when cultivated using citric acid as the sole available substrate [15]. *E. coli* displayed similar characteristics to *K. pneumoniae*. Lactic acid was entirely consumed for growth, and the amount of citric acid decreased by around half, which leads to the conclusion that citric acid was partially used for metabolism. The growth of *E. coli* in urine and the metabolic implications are well-studied and suggested Krebs cycle-based growth on ammonia, sulfate, creatinine, phosphate and amino acids [7,9,10,46].

The results for the qualitative analysis with ^1^H suggest metabolic differences between Gram-negative (utilizing lactic acid) and Gram-positive bacteria (utilizing citric acid) and, for all species, the utilization of peptone as an essential source for amino acids during growth. Notably, the amount of different growth compounds is reduced due to the proliferation of the bacteria, with the resultant release of trace amounts of other metabolites.

## 4. Discussion

The analysis of the e-tongue in infected AUM confirmed that the simple and robust e-tongue concept employed herein could be used to distinguish between infected and non-infected samples, where response changes could be related to metabolic activity of the bacteria. Importantly, detection of all investigated species was possible at clinically relevant bacterial concentrations, supporting that an e-tongue indeed could be used for rapid detection of UTI, where a response could be obtained within a few hours. Moreover, *K. pneumoniae* could be separated from the other species implying the potential for individual species classification within infected samples; however, it is crucial to exercise caution in extrapolating broad conclusions from limited studies. Factors such as growth phase and biological variation can influence the recorded response, which effects also may vary across bacterial species, as evidenced in this study. Given that bacteria are living organisms subject to intrinsic variability, the significance of biological replicates cannot be overstated. Their responses and growth patterns can differ even under identical conditions on different days. Furthermore, the task of distinguishing between bacterial species becomes increasingly complex when more species are introduced, potentially leading to similar response patterns among different species. Relaxing experimental conditions, particularly the control of bacterial density and growth phase, exacerbates this challenge.

Generally, the closer the metabolic similarities of the species of interest, the more complicated and challenging to separate. The analysis of bacterial growth in real urine is further complicated by the interplay between the bacterial metabolism and the human urine metabolome itself [68]. This makes the investigation challenging since all bacteria have individual growth characteristics, and many parameters need to be considered, like initial and changing pH over time, lack or excess of (preferred) substrate for growth, and aerobic or anaerobic conditions. Moreover, investigations of bacterial metabolites are only possible when analyzed in certain stages of their growth phase, e.g., log-phase relevant metabolites responsible for exponential growth [53].

Nevertheless, the ^1^H-NMR results suggested that certain Gram-positive and Gram-negative bacteria show different growth behavior in artificial urine. Hence, for *E. faecalis,* it was found that it cannot utilize lactic acid but is produced due to facultative anaerobic fermentation [61,69]. Moreover, citric acid was used as a substrate which led to the release of metabolites like acetic acid and ethanol [60,70]. In contrast, the two Gram-negative bacteria utilized lactic acid as a primary substrate for growth. This divergence underscores species-specific growth patterns driven by differing metabolisms (e.g., substrate utilization and urease production) and morphologies (e.g., coccus- vs. rod-shaped). Based on this, incorporating additional electrodes modified to improve their selectivity towards single metabolites could enhance the e-tongue’s discriminatory capabilities. Strategies may encompass enzyme incorporation, such as lactate dehydrogenase or ethanol dehydrogenase, or electrode modification with, e.g., conducting polymers or redox catalysts [71]. In addition, other types of measurements could also be included in the analysis, e.g., the inclusion of ion-selective electrodes. The combination of different sensor technologies in hybrid electronic tongues has been shown to improve performance [72]. However, incorporating more complicated sensors would require multistep modification protocols, reducing the simplicity of the e-tongue design as well as requiring more advanced data treatment, which was beyond the scope of the current study. Moreover, verifying and detecting one bacterium-specific metabolite would require more detailed molecular biological and microbiological investigations.

The e-tongue approach provides the possibility of a robust and rapid future sensor allowing for the detection and possibly also the classification of bacteria in urine. Transitioning from complex artificial solutions to authentic urine samples, future work would additionally include the evaluation of the sensor performance in human urine samples considering different sample collection times (morning vs. afternoon), gender disparities and dietary influences. Rigorous testing would be required to establish a dependable sensor response, given the marked variability inherent in human urine—a physiological fluid greatly influenced by factors such as dietary choices, hydration levels, and medication usage. Moreover, other species should be studied, for example, *Staphylococcus aureus* or *Proteus mirabilis*, which both have been found to be uropathogenic [23,24]. Developing a complete sensor array would allow for rapid decentralized diagnostics, leading to improved disease treatment with the possibility of individualized and precise (antibiotic) medication therapy [73].

## 5. Conclusions

In this study, we have successfully demonstrated the use of an e-tongue to discriminate between AUM samples devoid of bacterial growth and those exhibiting bacterial presence, all within the clinically significant threshold of 10^5^ CFU ml^−1^. The discernible variations in the recorded voltammograms can be attributed to changes in the AUM’s composition induced by bacterial metabolic activity. Furthermore, by utilizing flow cytometry, it was possible to quantitatively assess each distinct bacterial species at different time points during the incubation period. While subtle disparities emerged in the differential pulse voltammograms captured by the e-tongue when exposed to solutions containing different bacterial species, the application of PCA enabled the differentiation of *K. pneumoniae* from *E. coli* and *E. faecalis*. Moreover, the ^1^H-NMR results suggested that certain Gram-positive and Gram-negative bacteria show different growth behavior in artificial urine. Using this multifaceted approach enabled a deeper understanding of bacterial interactions within artificial urine, potentially paving the way for more precise diagnostics and treatment strategies.

## Figures and Tables

**Table 1:** Components of urine-mimicking AUM, adjusted to pH 6.4, adapted with permission from ref. [43] 2003, John Wiley and Sons.

Component	Quantity [g]
Uric acid	0.07
Peptone L37	1
Citric acid	0.4
Lactic acid	0.1
Urea	10
Creatinine	0.8
Yeast extract	0.005
Sodium bicarbonate	2.1
Calcium chloride	0.37
Sodium chloride	5.2
Iron II sulfate	0.0012
Magnesium sulfate	0.49
Sodium sulfate	3.2
Potassium dihydrogen phosphate	0.95
Di-potassium hydrogen phosphate	1.2
Ammonium chloride	1.3
Distilled water	Filled up to 1 L

**Table 2:** Incubation time vs. the measured concentration of each bacteria type at different times, as determined using flow cytometry.

Incubation Time (h)	*K. pneumoniae* Concentration (Cells/mL)	*E. faecalis* Concentration (Cells/mL)	*E. coli* Concentration (Cells/mL)
0	0.8 × 10^5^	0.6 × 10^5^	2.1 × 10^5^
1	2 × 10^5^	1.8 × 10^5^	3.8 × 10^5^
2	7.6 × 10^5^	4.8 × 10^5^	3.9 × 10^5^
3	2 × 10^6^	2.4 × 10^5^	5.5 × 10^5^
4	5.7 × 10^6^	1.4 × 10^5^	6 × 10^5^
5	1 × 10^7^	1.3 × 10^5^	5.3 × 10^5^

**Table 3:** Chemical shifts and peak intensities of the main components related to the recorded ^1^H NMR spectra of AUM and after 5 h of growth of *K. pneumoniae*, *E. faecalis* and *E. coli*.

Compound (ppm)	AUM (Black)	*K. pneumoniae* (Yellow)	*E. faecalis* (Green)	*E. coli* (Orange)
Lactic acid (d; 1.31, 1.32)	1233 1030	x	1629 1474	x
Citric acid (d; 2.50, 2.54, 2.65, 2.69)	4162 6859 6907 4034	1862 3518 3482 2049	x	1593 2469 2411 1427
Creatinine (s; 3.03 and 4.05)	57,212 20,801	18,743 17,631	34,434 13,077	25,369 19,200

## Data Availability

The data that support the findings of this study are available from the corresponding author upon request.

## Supplementary Materials

The following supporting information can be downloaded at https://www.mdpi.com/article/10.3390/bios13100916/s1. Figure S1: Flow cytometer *K. pneumoniae* results by displaying FITC-A (Fluorescein isothiocyanate) vs. cell count. Figure S2: Flow cytometer for *E. faecalis* results by displaying FITC-A (Fluorescein isothiocyanate) vs. cell count. Figure S3: Flow cytometer for *E. coli* results by displaying FITC-A (Fluorescein isothiocyanate) vs. cell count. Figure S4: Investigation of Pt (A), Pd (B) and Au (C) electrodes using DPV in PBS and PBS with each of the metabolically relevant compounds in AUM, using the same concentrations as used in AUM (see Table 1, MS): PBS (black), acetic acid (grey), uric acid (dark blue), peptone (green), citric acid (dark red), lactic acid (orange), urea (cyan), creatinine (light red). Figure S5: DPV comparing bacteria-infected AUM with the supernatant of the infected solution (solid = AUM; dashed = bacteria; dotted = supernatant): *K. pneumoniae* (top; *n* = 3), *E. faecalis* (middle; *n* = 3), *E. coli* (bottom; *n* = 3) and each electrode material (columns Pt (left) Pd (middle), Au (right)). Figure S6: Bacterial growth curves in AUM obtained by measuring the optical density for *K. pneumoniae* (A), *E. coli*, (B) and *E. faecalis* (C). Figure S7: Standard response currents (black = AUM; yellow *= K pneumoniae*; green = *E. faecalis*; orange = *E. coli*) for the investigated electrode materials Pt (A), Pd (B), and Au (C). Figure S8: ^1^H NMR spectrum of *E. coli* in AUM after 5 h of bacterial growth (reference peak, positioned at 4.05 ppm relative to TMS (0 ppm)). Figure S9: ^1^H NMR spectrum of *K. pneumoniae* in AUM after 5 h of bacterial growth (reference peak. positioned at 4.05 ppm relative to TMS (0 ppm)). Figure S10: ^1^H NMR spectrum of *E. faecalis* in AUM after 5 h of bacterial growth (reference peak positioned at 4.05 ppm relative to TMS (0 ppm)). Figure S11: ^1^H NMR spectrum of AUM (reference peak positioned at 4.05 ppm relative to TMS (0 ppm)). Figure S12: ^1^H NMR spectrum of peptone in D2O (reference peak positioned at 4.05 ppm relative to TMS (0 ppm)). Table S1: Score values related to the PCA of AUM and AUM incubated with three types of bacteria. Table S2: Score values related to the PCA of *K. pneumoniae* at different incubation times. Table S3: Score values related to the PCA of *E. faecalis* at different incubation times. Table S4: Score values related to the PCA of *E. coli* at different incubation times. Table S5: Score values related to the PCA of AUM and AUM incubated with three types of bacteria at different incubation times.
