# Supplementary material for: Bacteria-Infected Artificial Urine Characterization Based on a Combined Approach Using an Electronic Tongue Complemented with 1H-NMR and Flow Cytometry
Source: Biosensors (Basel). 2023 Oct 5;13(10):916. doi: 10.3390/bios13100916 (PMC10605348; doi:10.3390/bios13100916)
Supplement: Supplementary file 1 [file biosensors-13-00916-s001.zip › biosensors-2619832-supplementary.pdf]

Bacteria-infected artificial urine characterization based on a combined approach using an electronic tongue complemented with ^{1}H-NMR and flow cytometry

Carolin Psotta, Emelie J. Nilsson, Thomas Sjöberg and Magnus Falk*

Department of Biomedical Science. Faculty of Health and Society. Malmö University. Malmö 20506. Sweden
Biofilms-Research Center for Biointerfaces. Malmö University. Malmö 20506. Sweden

*corresponding author

Supporting material

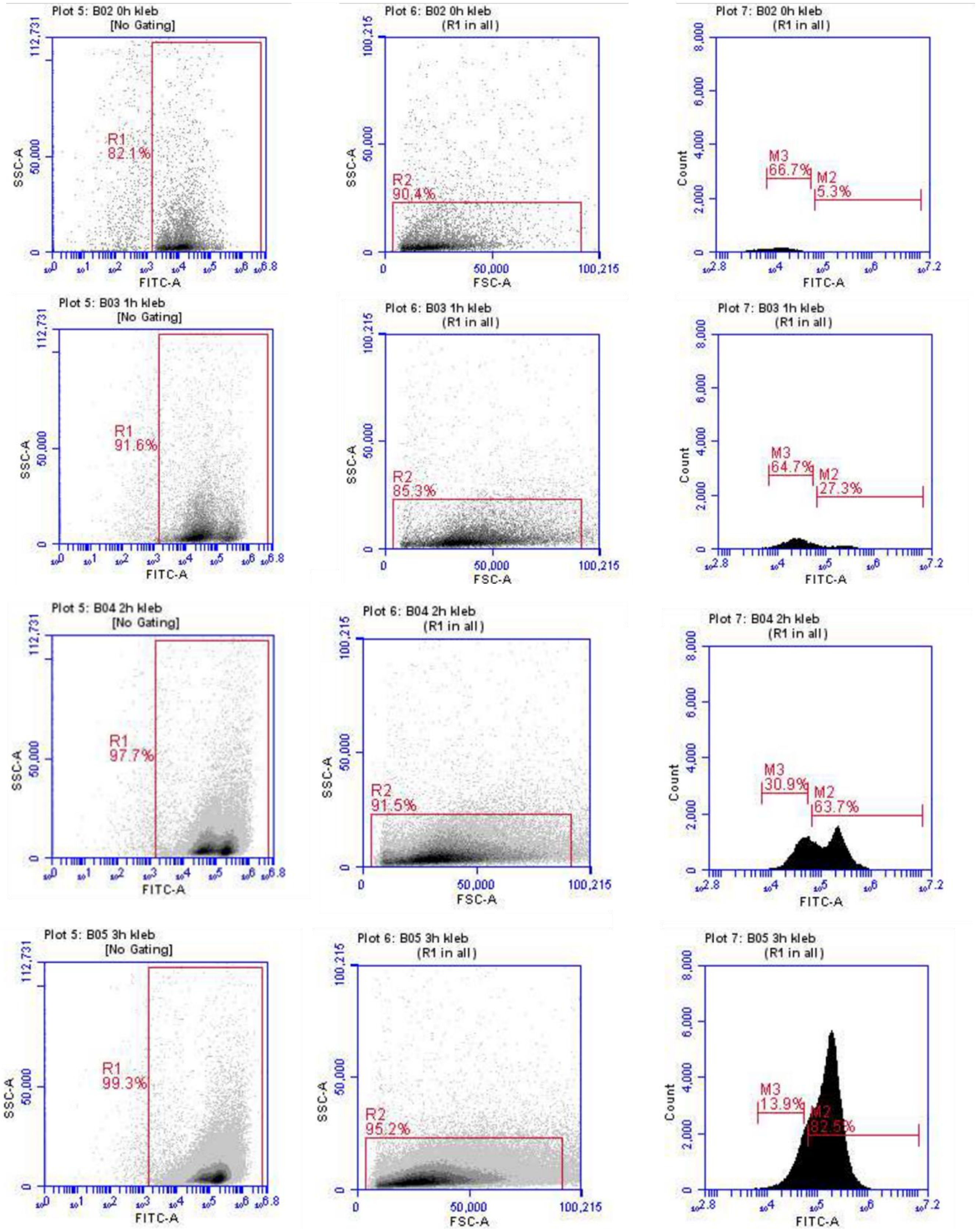
Plot 5: B02 0h kleb
[No Gating]
R1
82.1%
SSC-A
FITC-A
Plot 6: B02 0h kleb
(R1 in all)
R2
90.4%
SSC-A
FSC-A
Plot 7: B02 0h kleb
(R1 in all)
M3
66.7%
M2
5.3%
Count
FITC-A
Plot 5: B03 1h kleb
[No Gating]
R1
91.6%
SSC-A
FITC-A
Plot 6: B03 1h kleb
(R1 in all)
R2
85.3%
SSC-A
FSC-A
Plot 7: B03 1h kleb
(R1 in all)
M3
64.7%
M2
27.3%
Count
FITC-A
Plot 5: B04 2h kleb
[No Gating]
R1
97.7%
SSC-A
FITC-A
Plot 6: B04 2h kleb
(R1 in all)
R2
91.5%
SSC-A
FSC-A
Plot 7: B04 2h kleb
(R1 in all)
M3
30.9%
M2
63.7%
Count
FITC-A
Plot 5: B05 3h kleb
[No Gating]
R1
99.3%
SSC-A
FITC-A
Plot 6: B05 3h kleb
(R1 in all)
R2
95.2%
SSC-A
FSC-A
Plot 7: B05 3h kleb
(R1 in all)
M3
13.9%
M2
82.5%
Count
FITC-A

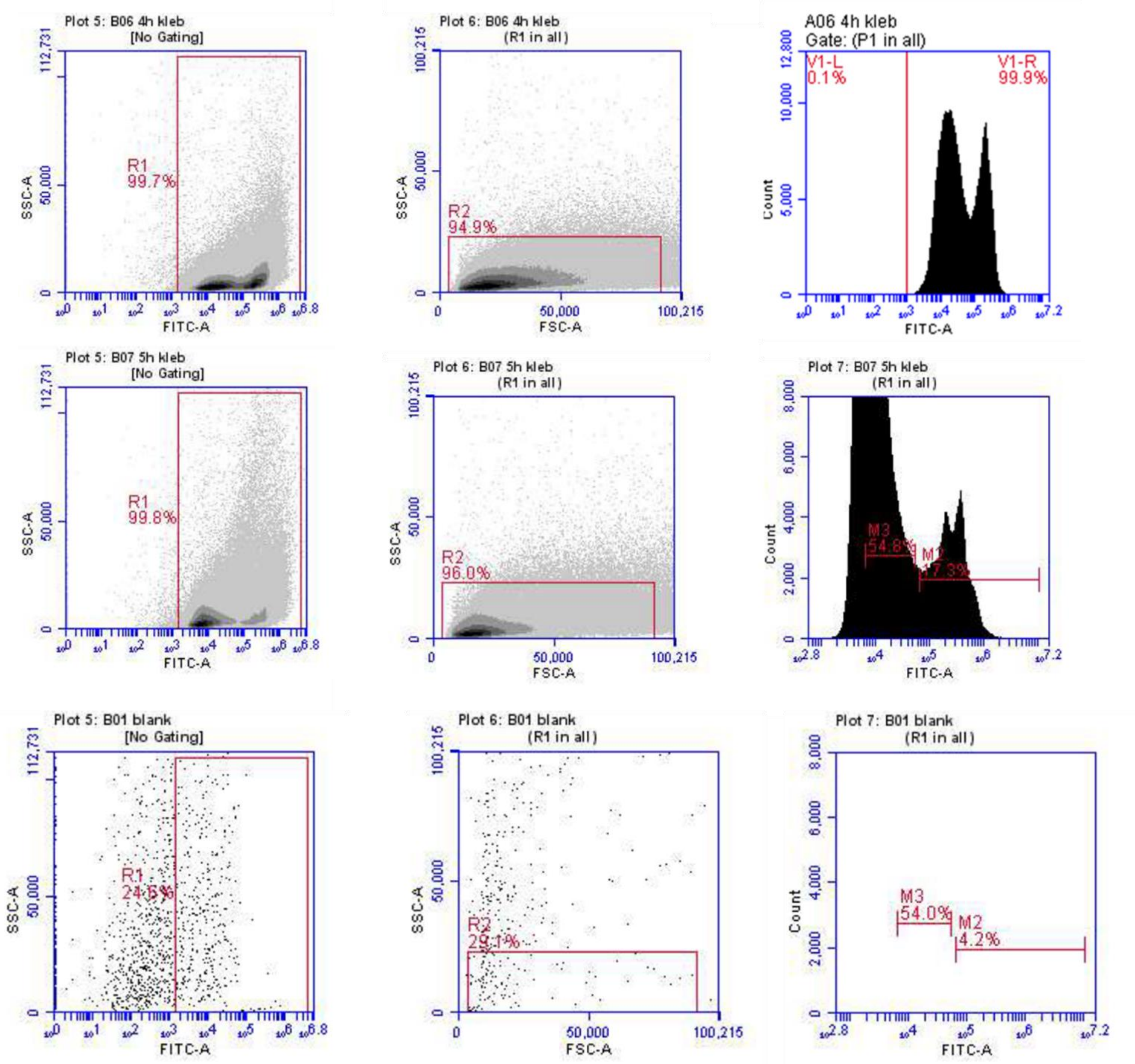

Supporting Figure S1: Flow cytometry results for *K.pneumoniae,* displaying FITC-A (Fluorescein isothiocyanate) *vs.* cell count.

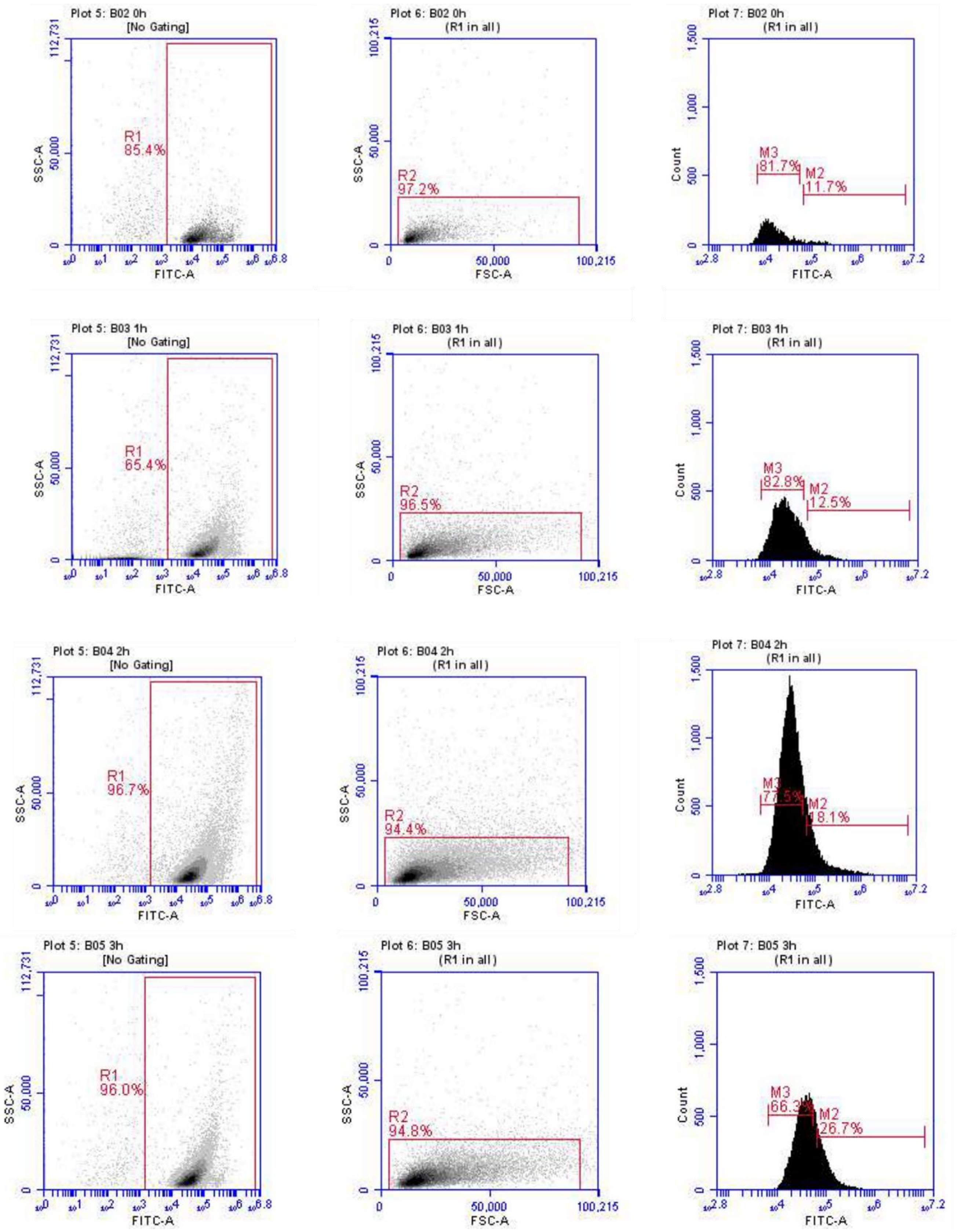
Plot 5: B02 0h
[No Gating]
R1
85.4%
SSC-A
FITC-A
Plot 6: B02 0h
(R1 in all)
R2
97.2%
SSC-A
FSC-A
Plot 7: B02 0h
(R1 in all)
M3
81.7%
M2
11.7%
Count
FITC-A
Plot 5: B03 1h
[No Gating]
R1
65.4%
SSC-A
FITC-A
Plot 6: B03 1h
(R1 in all)
R2
96.5%
SSC-A
FSC-A
Plot 7: B03 1h
(R1 in all)
M3
82.8%
M2
12.5%
Count
FITC-A
Plot 5: B04 2h
[No Gating]
R1
96.7%
SSC-A
FITC-A
Plot 6: B04 2h
(R1 in all)
R2
94.4%
SSC-A
FSC-A
Plot 7: B04 2h
(R1 in all)
M3
77.5%
M2
18.1%
Count
FITC-A
Plot 5: B05 3h
[No Gating]
R1
96.0%
SSC-A
FITC-A
Plot 6: B05 3h
(R1 in all)
R2
94.8%
SSC-A
FSC-A
Plot 7: B05 3h
(R1 in all)
M3
66.3%
M2
26.7%
Count
FITC-A

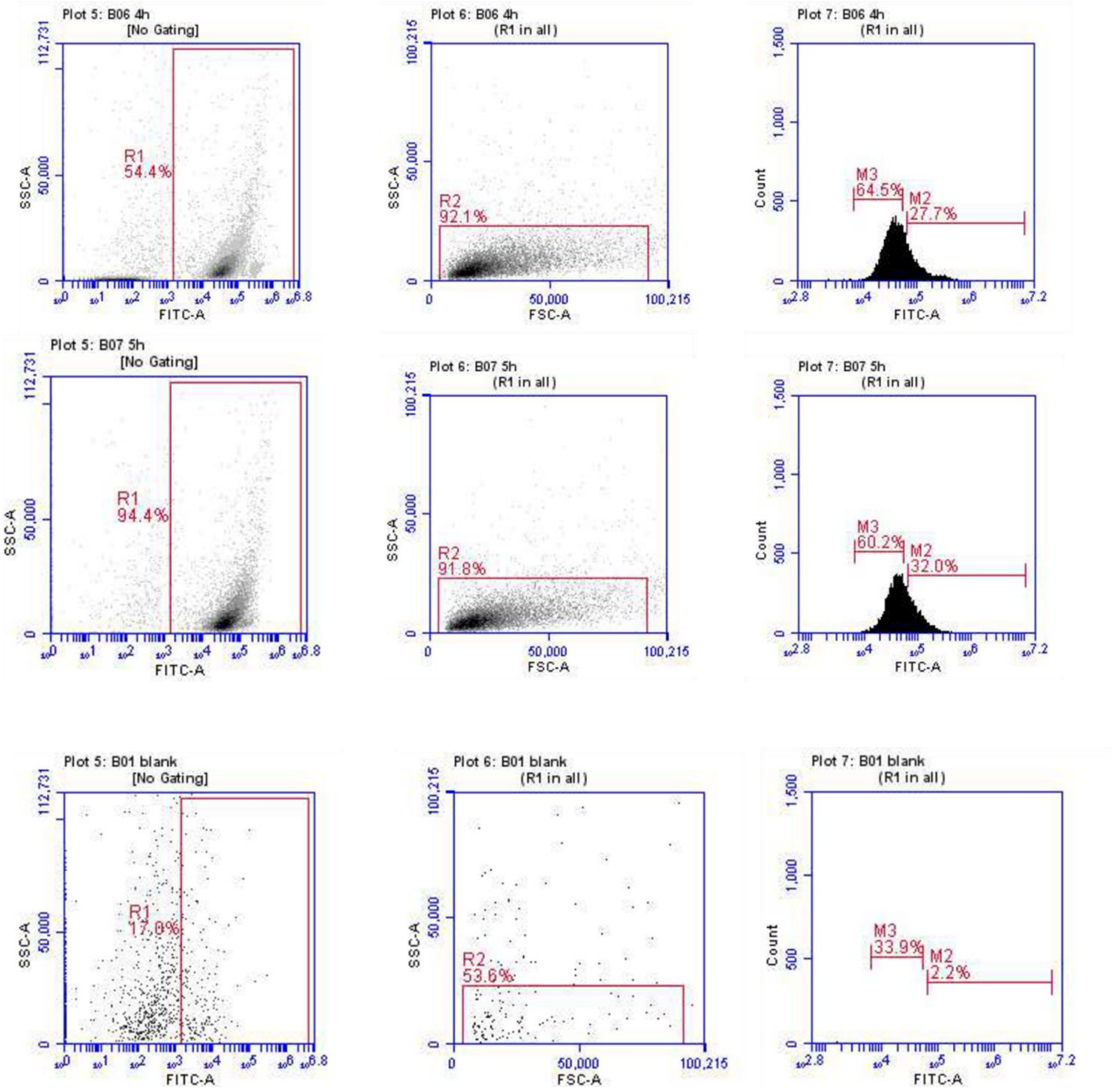

Supporting Figure S2: Flow cytometry results for *E.faecalis,* displaying FITC-A (Fluorescein isothiocyanate) *vs.* cell count.

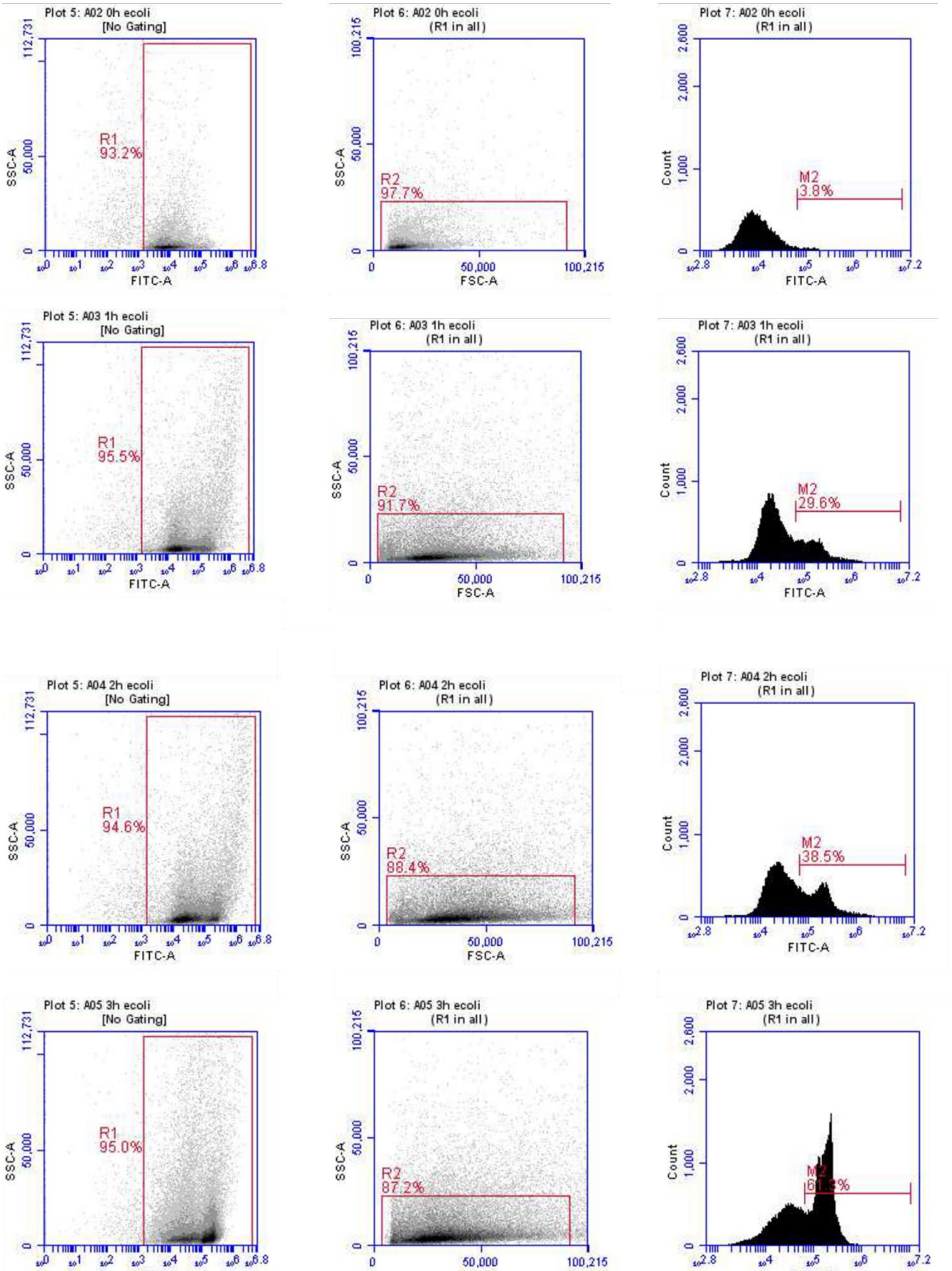
Plot 5: A02 0h ecoli
[No Gating]
R1
93.2%
SSC-A
FITC-A
Plot 6: A02 0h ecoli
(R1 in all)
R2
97.7%
SSC-A
FSC-A
Plot 7: A02 0h ecoli
(R1 in all)
M2
3.8%
Count
FITC-A
Plot 5: A03 1h ecoli
[No Gating]
R1
95.5%
Plot 6: A03 1h ecoli
(R1 in all)
R2
91.7%
Plot 7: A03 1h ecoli
(R1 in all)
M2
29.6%
Plot 5: A04 2h ecoli
[No Gating]
R1
94.6%
Plot 6: A04 2h ecoli
(R1 in all)
R2
88.4%
Plot 7: A04 2h ecoli
(R1 in all)
M2
38.5%
Plot 5: A05 3h ecoli
[No Gating]
R1
95.0%
Plot 6: A05 3h ecoli
(R1 in all)
R2
87.2%
Plot 7: A05 3h ecoli
(R1 in all)
M2

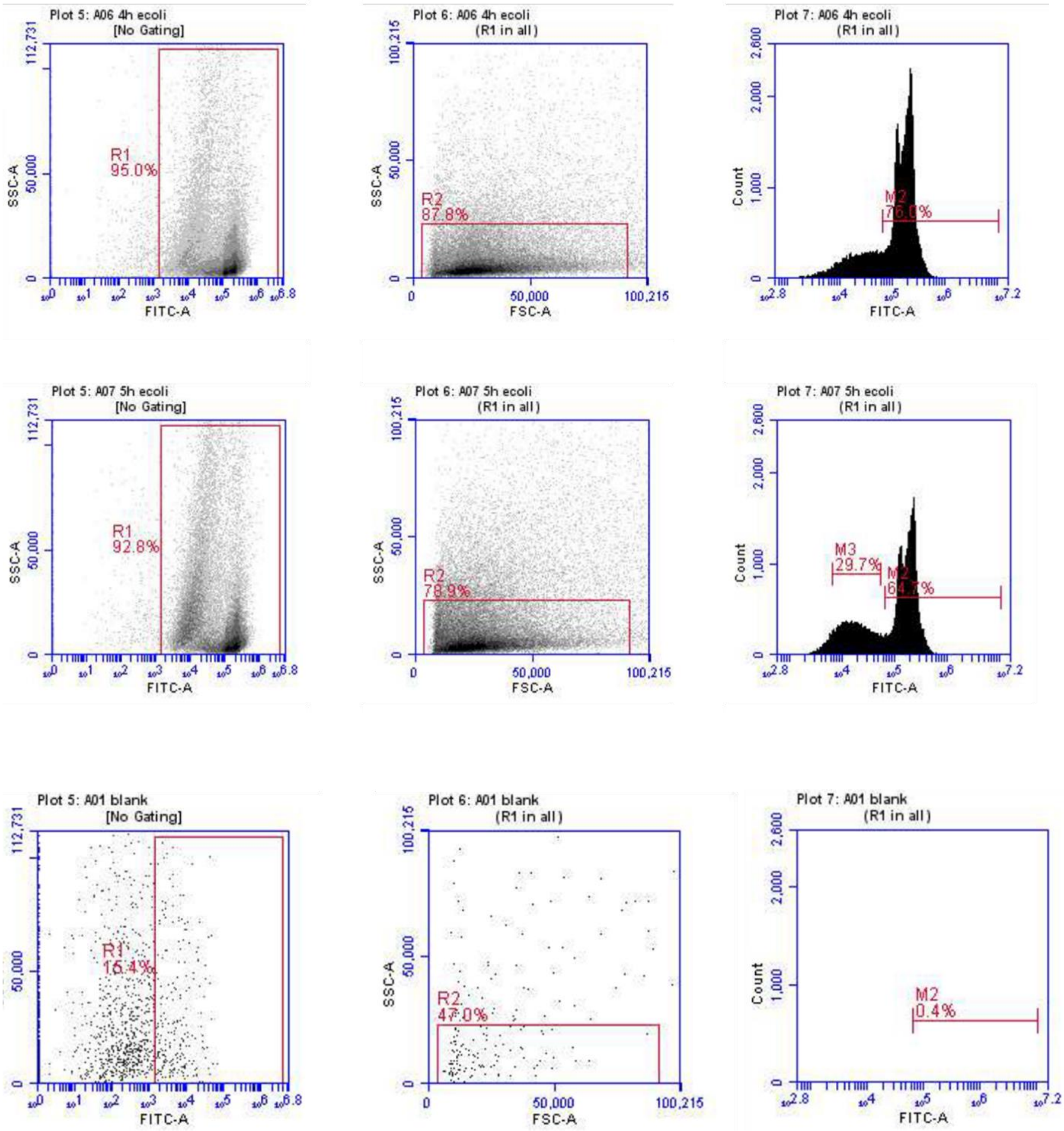

Supporting Figure S3: Flow cytometry results for *E.coli,* displaying FITC-A (Fluorescein isothiocyanate) *vs.* cell count.

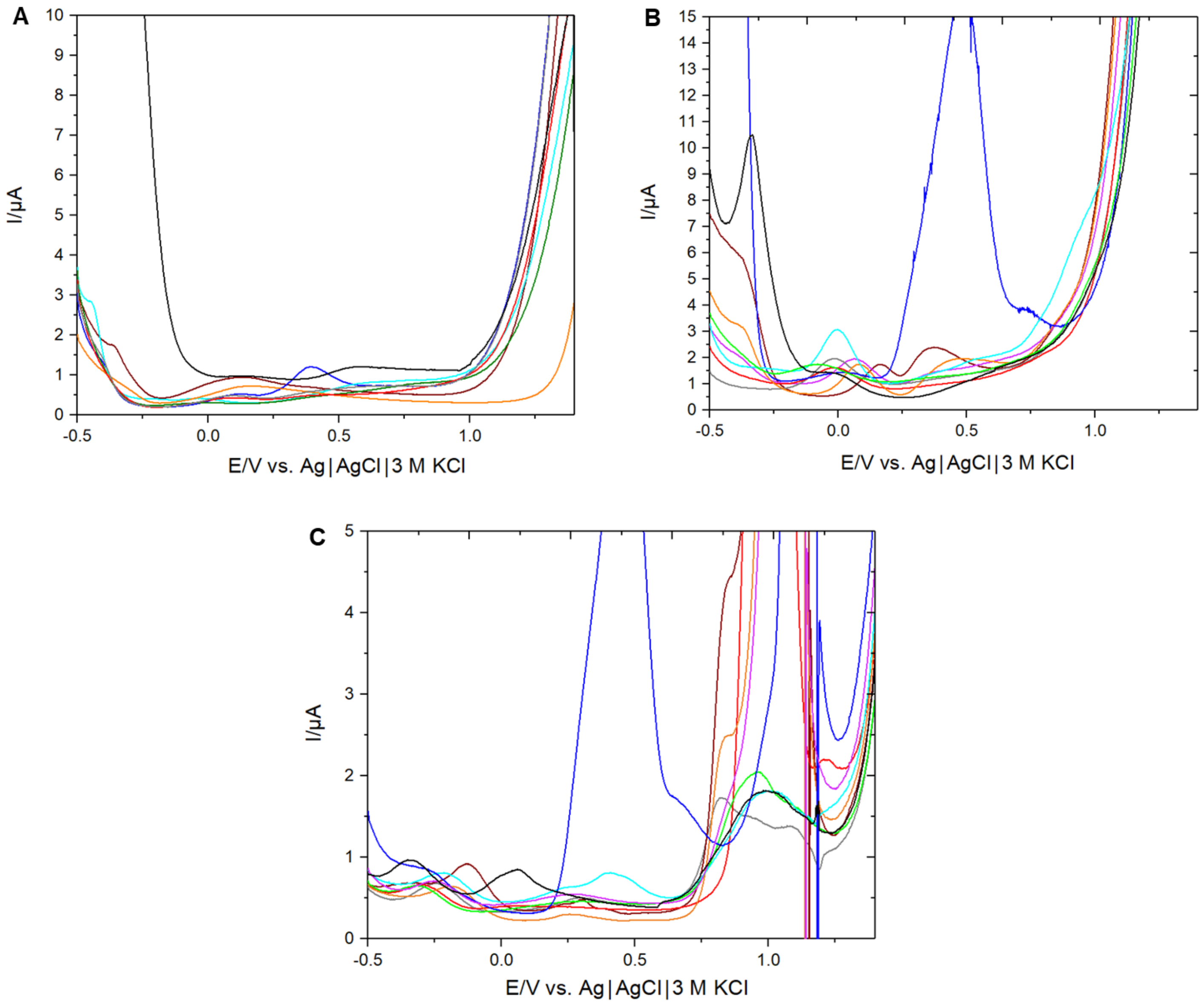

Supporting Figure S4: Investigation of Pt (**A**), Pd (**B**) and Au (**C**) electrodes using DPV in PBS and PBS with each of the metabolically relevant compounds in AUM, using the same concentrations as used in AUM (see Table 1, MS): PBS (black), acetic acid (grey). uric acid (dark blue). peptone (green). citric acid (dark red). lactic acid (orange). urea (cyan). creatinine (light red).

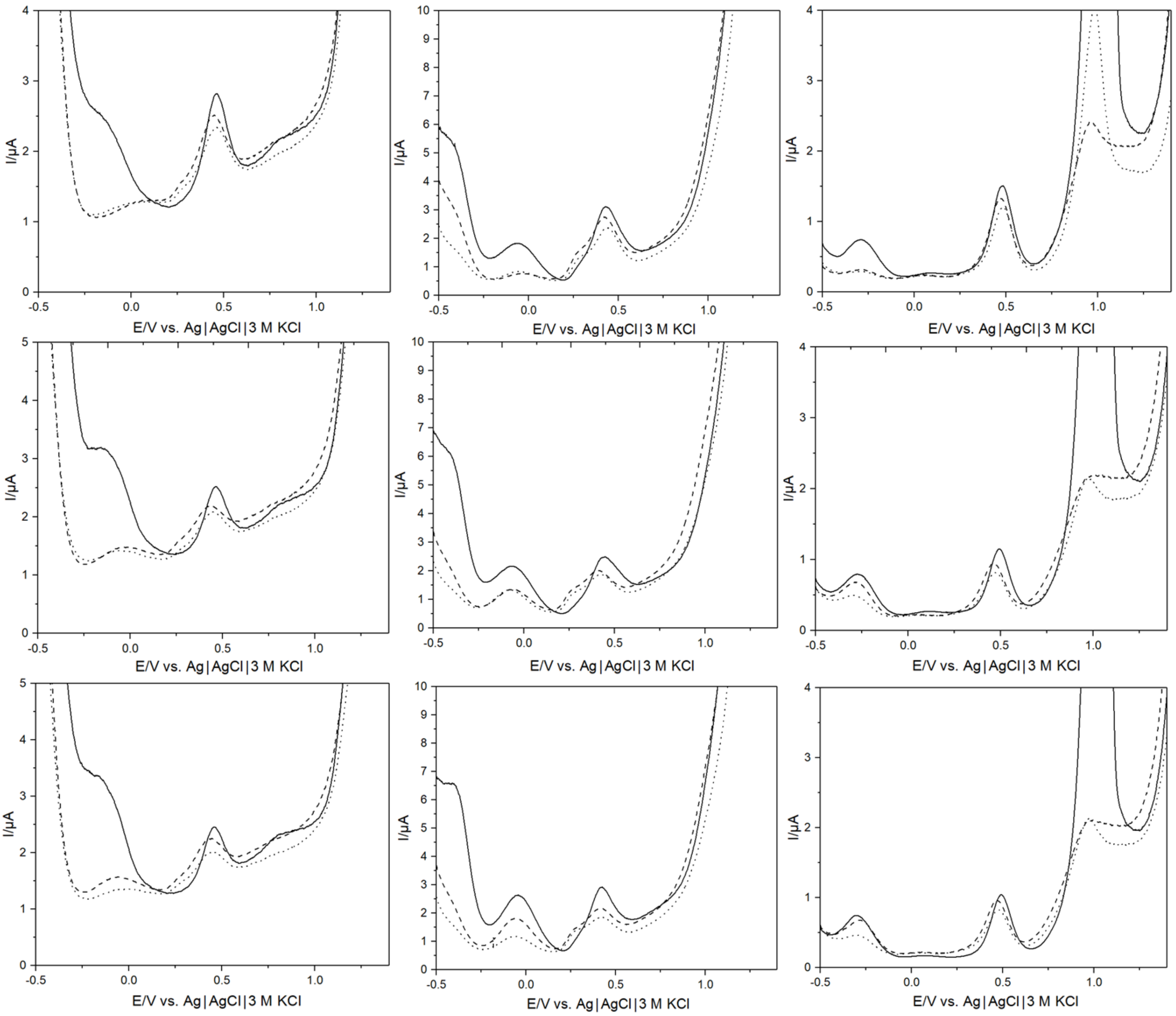

Supporting Figure S5: DPV comparing bacteria-infected AUM with the supernatant of the infected solution (solid = AUM; dashed = bacteria; dotted = supernatant): *K.pneumoniae* (top; n = 3), *E.faecalis* (middle; n = 3), *E.coli* (bottom; n = 3) and each electrode material (columns Pt (left) Pd (middle), Au (right)).

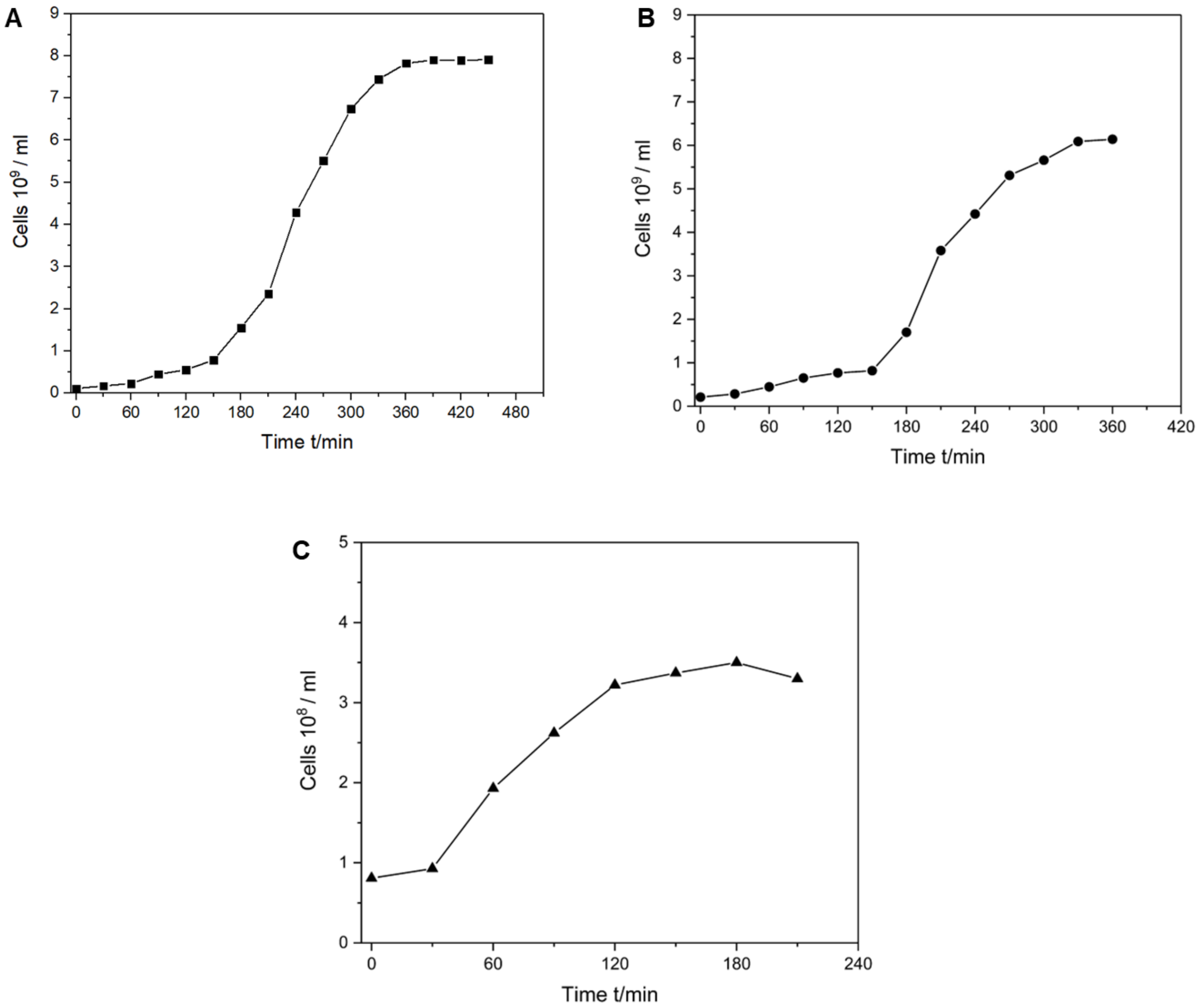

Supporting Figure S6: Bacterial growth curves in AUM obtained by measuring the optical density for *K.pneumoniae* (A), *E.coli.* (B) and *E.faecalis* (C)

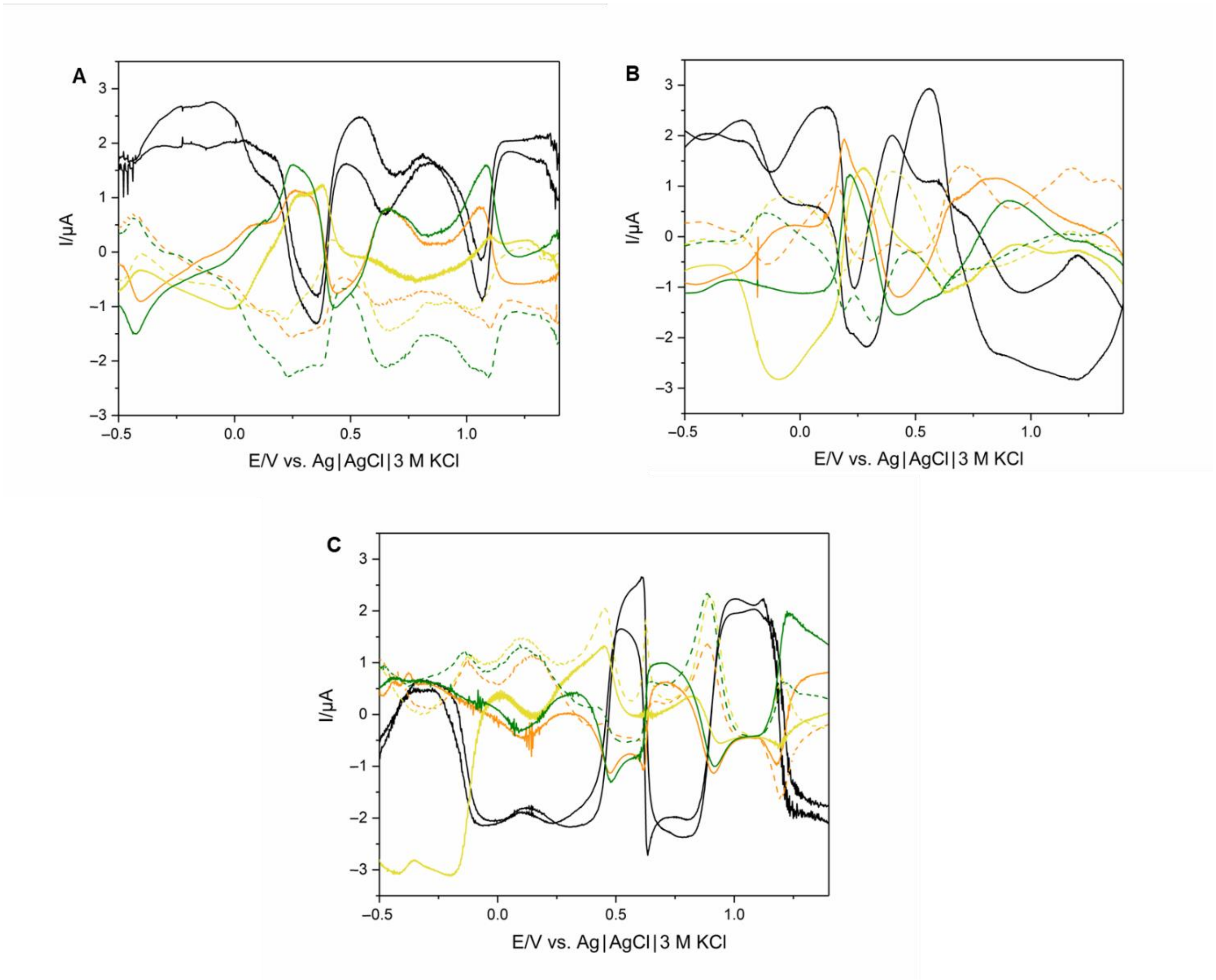

Supporting Figure S7: Normalized current response (subtracting the mean and dividing with the standard deviation) at each potential using the whole data set used for Fig. 4, (black = AUM; yellow = *K.pneumoniae*, green = *E.faecalis*, orange = *E.coli,* 1 h = dashed, 5 h = solid)) for the investigated electrode materials Pt (A), Pd (**B**), and Au (**C**)

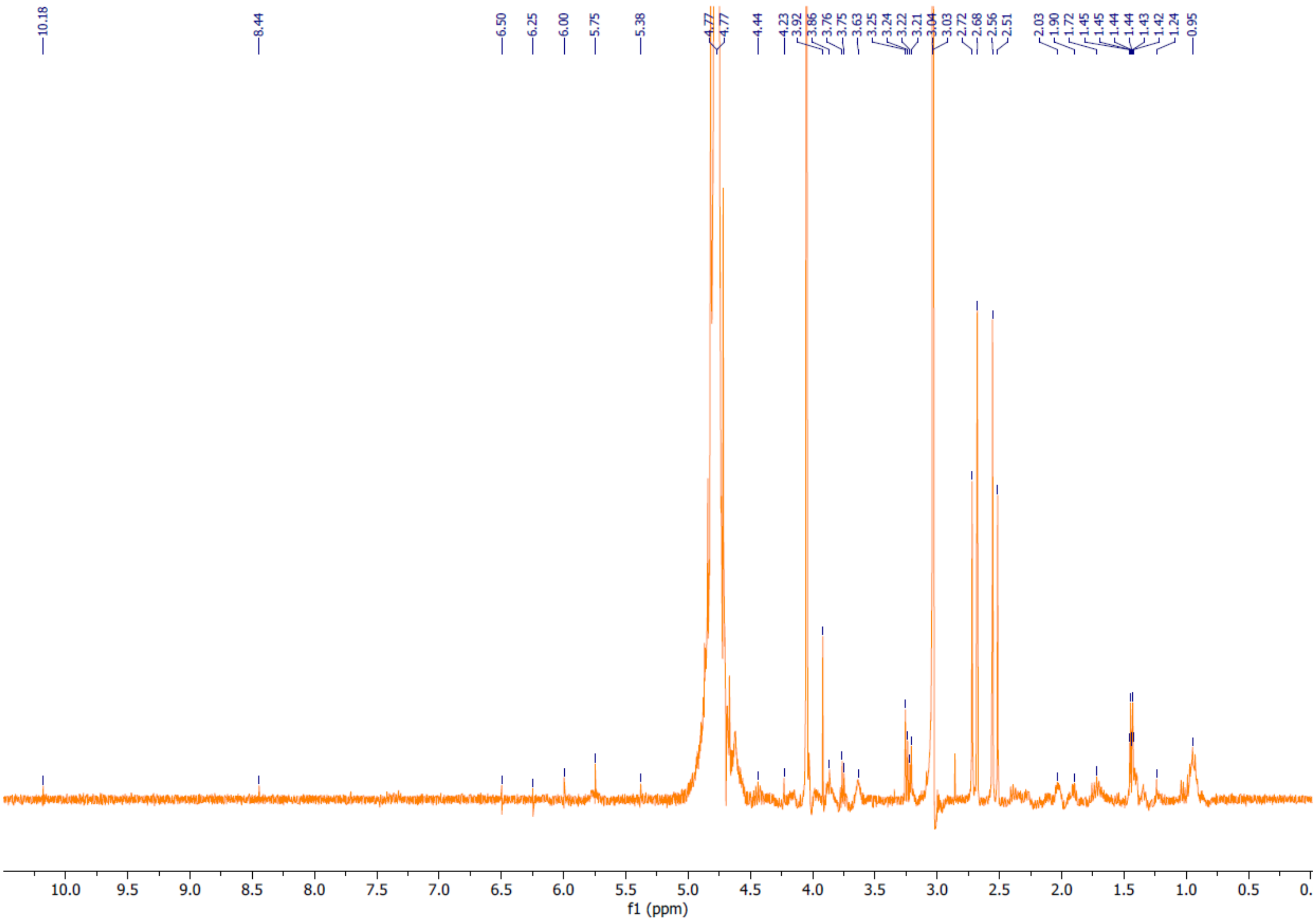

Supporting Figure S8: ^{1}H NMR spectrum of *E.coli* in AUM after 5h of bacterial growth (reference peak. positioned at 4.05 ppm relative to TMS (0 ppm)).

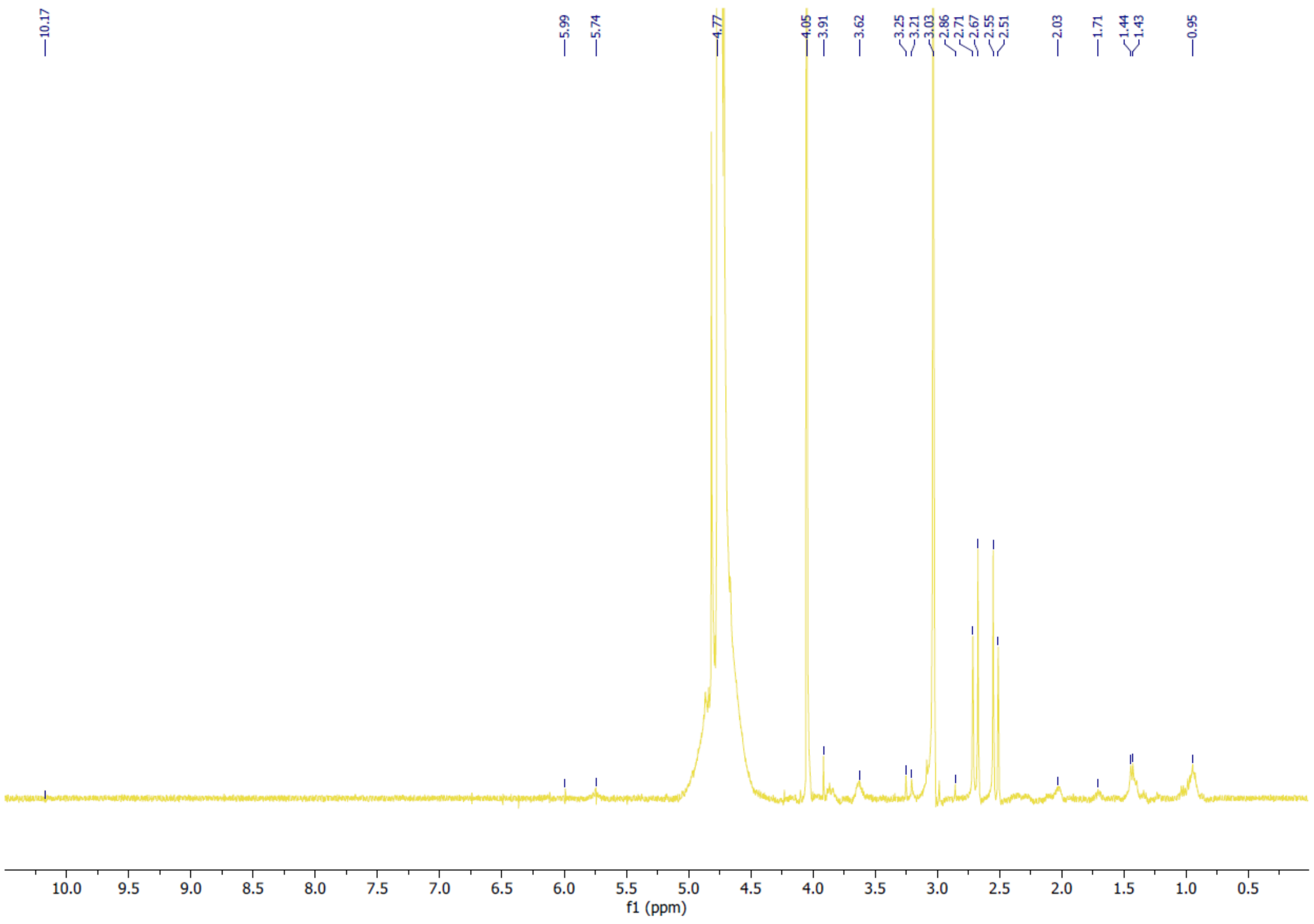

Supporting Figure S9: ^{1}H NMR spectrum of *K.pneumoniae* in AUM after 5h of bacterial growth (reference peak. positioned at 4.05 ppm relative to TMS (0 ppm)).

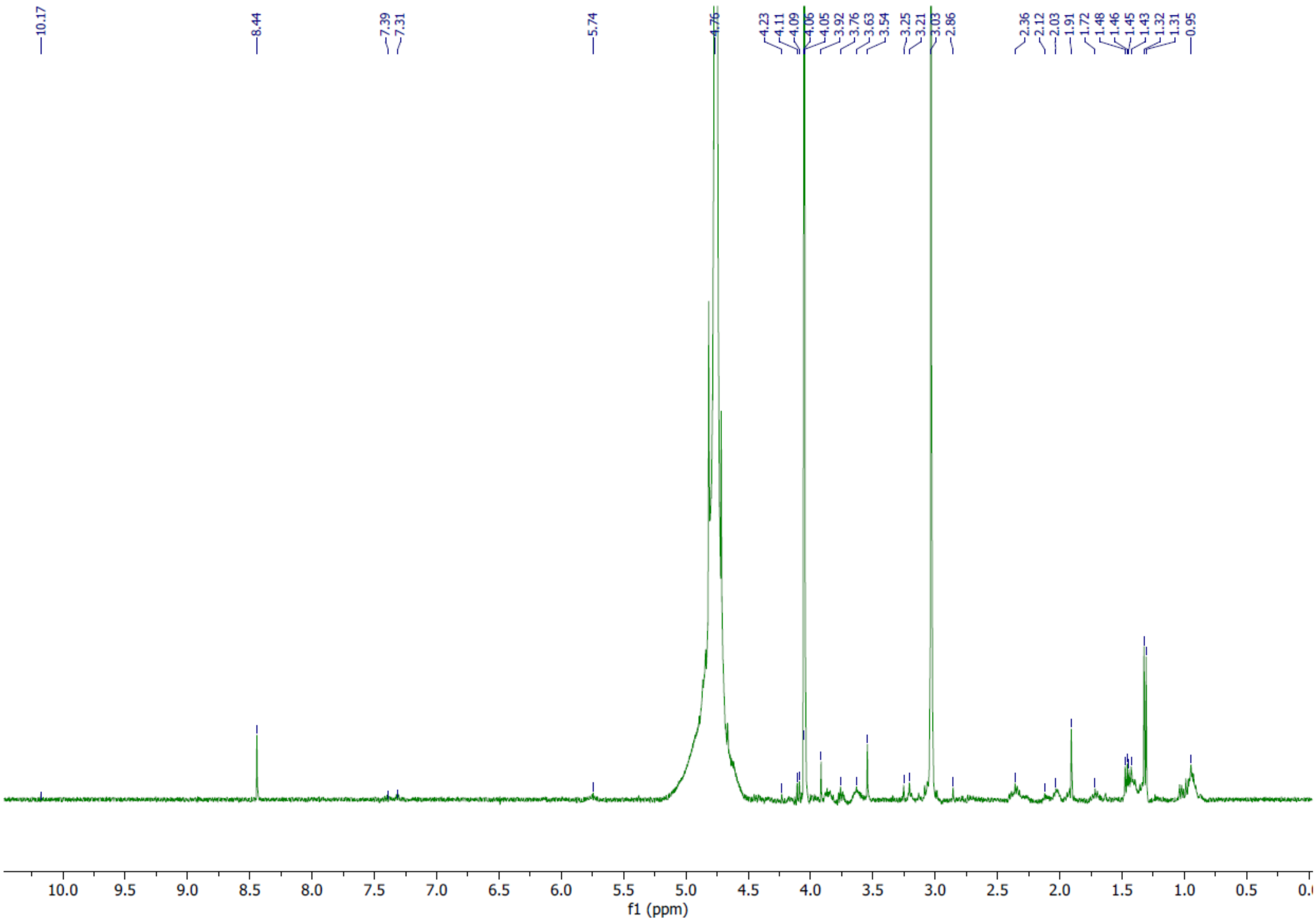

Supporting Figure S10: ^{1}H NMR spectrum of *E.faecalis* in AUM after 5h of bacterial growth (reference peak. positioned at 4.05 ppm relative to TMS (0 ppm)).

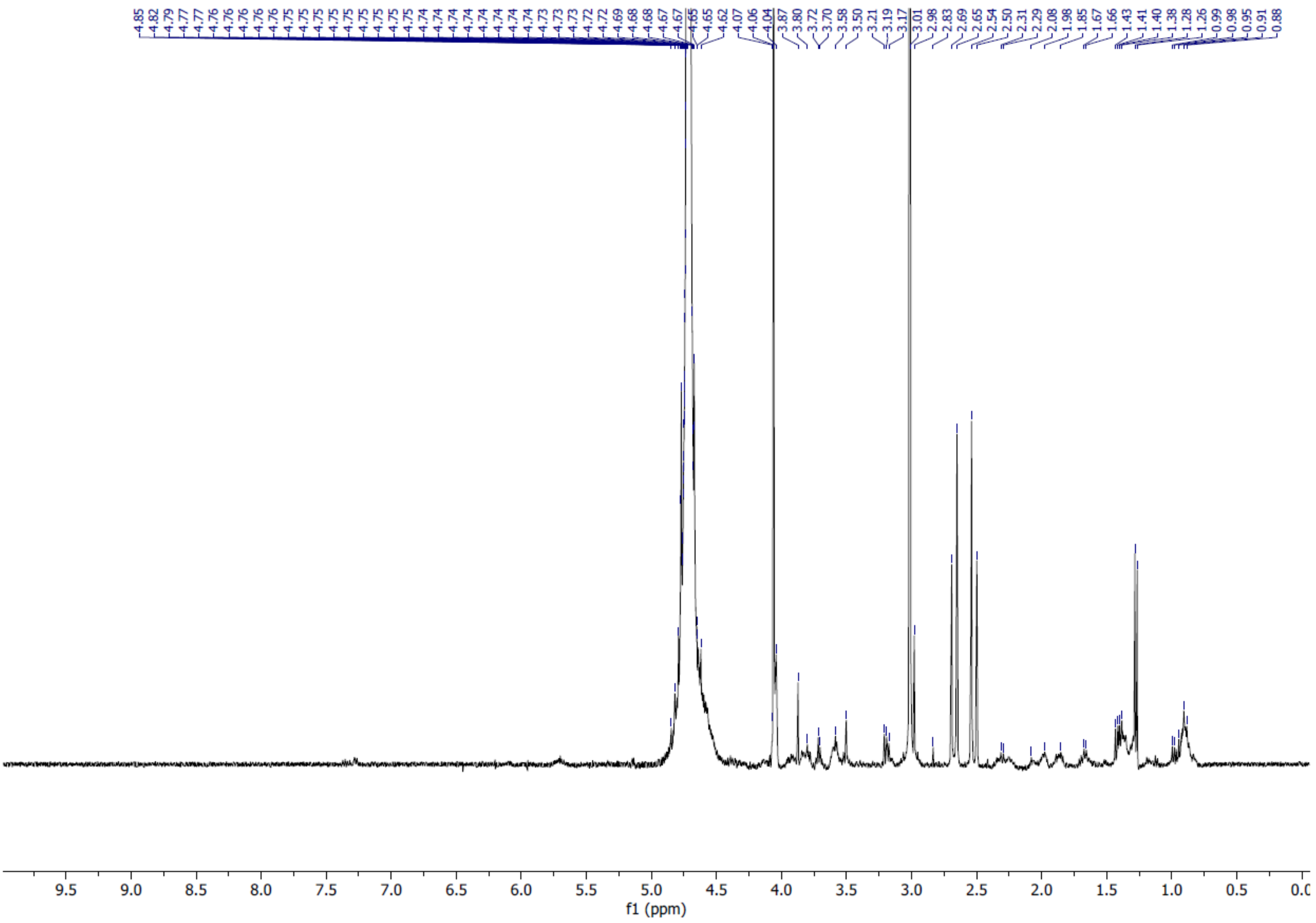

Supporting Figure S11: ^{1}H NMR spectrum of AUM (reference peak. positioned at 4.05 ppm relative to TMS (0 ppm)).

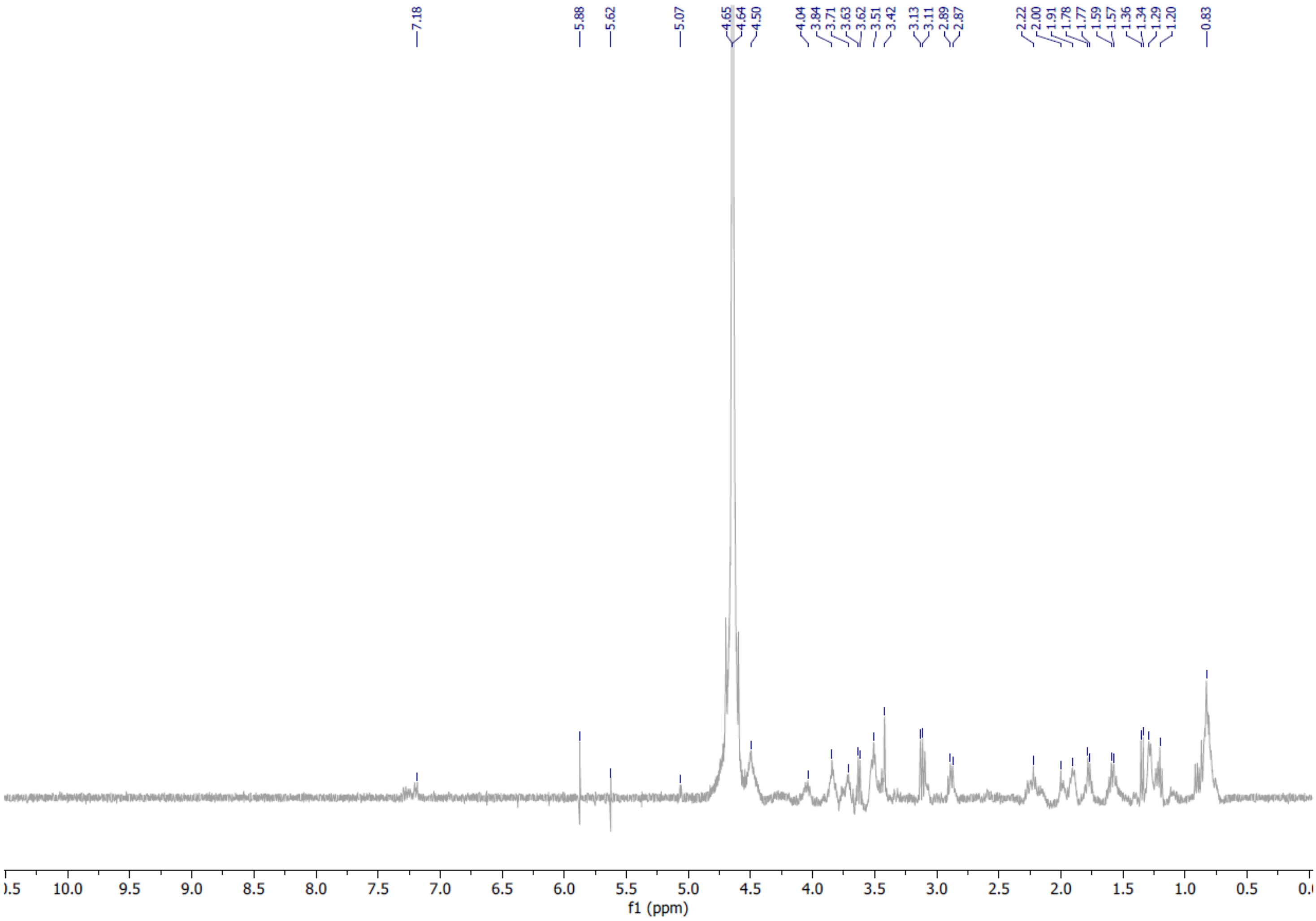

Supporting Figure S12: ^{1}H NMR spectrum of peptone in D_2O (reference peak. positioned at 4.05 ppm relative to TMS (0 ppm)).

Supporting Table S1: Score values related to the PCA of AUM and AUM incubated with three types of bacteria.

Sample name	Component 1	Component 2	Component 3	Component 4
ECfeac 1	0.174	0.554	-0.586	0.034
ECfeac 2	0.384	0.089	0.831	-0.121
ECfaec 3	0.006	-0.171	-0.267	-0.939
Ecoli 1	0.332	0.860	-0.113	0.225
Ecoli 2	0.311	0.875	-0.096	0.212
Ecoli 3	0.367	0.291	0.800	-0.039
Kleb 1	0.664	-0.347	-0.243	0.439
Kleb2	0.724	-0.500	0.025	0.345
Kleb 3	0.773	-0.463	-0.071	0.300
Aum1	-0.910	0.009	0.240	0.276
Aum2	-0.885	0.038	0.187	0.328
Aum 3	-0.844	-0.235	-0.167	0.370

Supporting Table S2: Score values related to the PCA of *K.pneumoniae* at different incubation times.

Sample name	Component 1	Component 2
Aum1	-.906	.336
Aum2	-.945	.106
Aum3	-.806	-.098
1h	.712	-.684
2h	.948	-.241
3h	.925	.205
4h	.884	.439
5h	.733	.633

Supporting Table S3: Score values related to the PCA of *E.faecalis* at different incubation times

Sample name	Component 1	Component 2
Aum1	-.902	.314
Aum2	-.945	.093
Aum3	-.842	.018
1h	.705	-.697
2h	.924	-.134
3h	.970	.146
4h	.792	.512
5h	.740	.613

Supporting Table S4: Score values related to the PCA of *E.coli* at different incubation times.

Sample name	Component 1	Component 2
Aum1	-.893	.240
Aum2	-.932	.150
Aum3	-.902	-.105
1h	.795	-.534
2h	.903	-.350
3h	.748	.344
4h	.880	.357
5h	.767	.559

Supporting Table S5: Score values related to the PCA of AUM and AUM incubated with three types of bacteria at different incubation times.

Sample name	Component 1	Component 2	Component 3	Component 4
Aum 1	-.946	-.238	.097	-.120
Aum2	-.914	-.324	-.060	-.110
Aum3	-.834	-.419	-.160	.089
Ecoli 1h	.595	-.104	-.628	.408
Ecoli 2h	.716	-.221	-.411	.458
Ecoli 3h	.525	-.428	.178	.383
Ecoli 4h	.718	-.387	.304	.375
Ecoli 5h	.591	-.353	.611	.193
Kleb 1h	.235	.576	-.691	-.202
Kleb2h	.162	.883	-.285	.010
Kleb 3h	.018	.918	.238	.221
Kleb 4h	.013	.868	.419	.211
Kleb 5h	-.092	.768	.433	.154
ECfeac 1h	.491	.041	-.788	-.301
ECfeac 2h	.765	-.096	-.362	-.421
ECfaec 3h	.889	-.066	.091	-.380
ECfaec 4h	.588	-.072	.605	-.408
ECfaec 5h	.494	-.038	.740	-.390
